# Generation of hiPSC-Derived Brain Microvascular Endothelial Cells Using Directed Differentiation and Transcriptional Reprogramming

**DOI:** 10.1161/ATVBAHA.125.323397

**Published:** 2025-11-25

**Authors:** Aomeng Cui, Ronak Patel, Patrick Bosco, Uğur Akcan, Emily Richters, Paula Barrilero Delgado, Dritan Agalliu, Andrew A. Sproul

**Affiliations:** 1 Department of Neurology (A.C., U.A., D.A.), Columbia University Irving Medical Center, New York, NY.; 2 Taub Institute for Research on Alzheimer’s Disease and the Aging Brain (R.P., P.B., E.R., A.A.S.), Columbia University Irving Medical Center, New York, NY.; 3 Department of Pathology and Cell Biology (D.A., A.A.S.), Columbia University Irving Medical Center, New York, NY.; 4 Francisco de Vitoria University, Madrid, Spain (P.B.D.).

**Keywords:** astrocytes, blood-brain barrier, endothelial cells, pericytes, tight junctions

## Abstract

**BACKGROUND:**

Modeling the human blood-brain barrier (BBB) is limited by the lack of robust protocols to generate induced pluripotent stem cell (iPSC)–derived brain microvascular endothelial cells (BMECs). Current methods generate cells that do not fully recapitulate key BMEC functions or the brain endothelial transcriptome identity.

**METHODS:**

To address this gap, we combined directed differentiation of human iPSCs into BBB-primed endothelial cells with overexpression of FOXF2 (forkhead box F2) and ZIC3 (zic family zinc finger 3), transcription factors critical for BMEC identity, to generate reprogrammed BMECs (rBMECs) from 3 iPSC lines. We performed immunofluorescence, functional analyses, and bulk RNA sequencing to characterize these cells. We cocultured rBMECs with iPSC-derived astrocytes and pericytes in the MIMETAS microfluidics platform to assess how 3-dimensional culture influences their BBB properties. Finally, we generated rBMECs expressing familial Alzheimer disease mutation *APP V717I* to elucidate how this genetic variant affects barrier properties compared with exposure to oAβ42 (oligomeric amyloid-β [1-42] peptide).

**RESULTS:**

Transcriptomic and functional analyses show that rBMECs express a subset of the BBB transcriptome and exhibit stronger paracellular barrier properties, lower caveolar-mediated transport, and comparable PGP (P-glycoprotein) activity compared with primary human BMECs. rBMECs interact with human iPSC–derived pericytes and astrocytes to form a 3D neurovascular system in the MIMETAS microfluidics platform with robust BBB properties. Finally, *APP V717I* rBMECs show decreased barrier integrity and upregulation of inflammatory markers. In contrast, treatment of control rBMECs with oAβ42 increases inflammatory markers, but does not alter barrier integrity.

**CONCLUSIONS:**

This protocol generates rBMECs with strong BBB properties and a brain-specific transcriptome signature. In addition, the iPSC-derived 3D neurovascular unit system shows some similar properties to the in vivo human BBB. Finally, familial Alzheimer disease mutation *APP V717I* alters several BBB-related properties of rBMECs and their inflammatory state, independent of Aβ42 (amyloid-β [1-42] peptide).

**What Are the Clinical Implications?**

Modeling the human blood-brain barrier (BBB) in vitro to elucidate neurological disease mechanisms and develop potential therapeutics remains limited by the lack of robust protocols to generate induced pluripotent stem cell–derived brain microvascular endothelial cells (BMECs). Existing methods generate cells that do not fully recapitulate key BMEC functions or the brain endothelial transcriptome identity. To address this gap, we have generated reprogrammed BMECs (rBMECs) through combining directed differentiation into BBB-primed endothelial cells with overexpression of 2 transcription factors critical for BMEC identity: FOXF2 (forkhead box F2) and ZIC3 (zic family zinc finger 3). rBMECs express a subset of the BBB transcriptome and exhibit stronger BBB properties compared with primary human BMECs. rBMECs interact with human induced pluripotent stem cell–derived pericytes and astrocytes to form a 3D neurovascular system in the MIMETAS microfluidics platform with robust BBB properties. rBMECs carrying familial Alzheimer disease mutation *APP V717I* show decreased barrier integrity and upregulation of inflammatory markers. Therefore, rBMECs closely resemble brain ECs at both the molecular and functional levels, suggesting that they may be utilized to study pathogenic mechanisms of neurological disease and to develop efficient methods for drug delivery to the central nervous system.

The blood-brain barrier (BBB), formed by brain microvascular endothelial cells (BMECs), is critical for maintaining central nervous system (CNS) homeostasis. BBB function is impaired in several neurological disorders, and its presence hinders the delivery of therapeutics into the CNS.^1^ BMECs interact with pericytes and astrocytes via the extracellular matrix (ECM) to form the neurovascular unit (NVU). These interactions are critical to establish several BBB properties including: (1) the presence of tight junctions (TJs) that restrict paracellular flux; (2) reduction in transcellular transport (transcytosis) due to few caveolae; (3) expression of specialized transporters that regulate the passage of nutrients and restrict the passage of drugs; and (4) low expression of leukocyte adhesion molecules (LAMs) that mediate immune cell trafficking into the CNS.^2–6^ Rodent models, although useful for studying mechanisms of CNS diseases including those with BBB impairment, do not capture patient heterogeneity in multifactorial neurological disorders, and their NVU cells differ from those of humans at the molecular level.^7–9^ These discrepancies necessitate the development of in vitro human NVU systems that have robust molecular and functional BBB properties but are also convenient and reproducible to isolate specific mechanisms underlying neurological diseases and develop safe, effective therapeutics to enter the CNS.^10–13^

**See cover image**

Primary human BMECs (HBMECs) are scarce, heterogeneous, and dedifferentiate into generic endothelial cells (ECs) upon passaging.^14–16^ Human induced pluripotent stem cells (hiPSCs) are a promising alternative to generate ECs due to their scalability and ability to model various pathological conditions. Over the past decade, several protocols have been developed to model brain ECs, termed induced pluripotent stem cell (iPSC)–derived BMECs (iBMECs), with high transendothelial electrical resistance (TEER) and expression of TJ proteins.^17–23^ While iBMECs offered some initial insights into disease mechanisms, as these cells exhibit a subset of EC functional properties (ie, strong barrier properties and expression of a subset of transporters), we recently showed that iBMECs have an epithelial cell identity at the transcriptome level.^24^ Moreover, iBMECs upregulate LAMs (leukocyte adhesion molecules) inconsistently in response to inflammatory stimuli, limiting their ability to model disease.^25^ Additional approaches have used extended passaging of endothelial progenitor cells (EPCs) to generate ECs. Mature EPC-ECs demonstrate good barrier properties but limited response to inflammatory stimuli.^25,26^ Finally, treatment of iPSC-derived ECs with a defined small molecule cocktail can enhance certain BBB properties without inducing expression of brain-specific identity markers.^27^ Thus, understanding how the human BBB changes in pathological states remains challenging, in part, due to the limitations of current methods to generate bona fide brain ECs.

Comparisons of transcriptomes and chromatin accessibility of ECs from various organs have identified several transcription factors essential for BMEC identity including FOXF2, FOXQ1 (forkhead box Q1), LEF1 (lymphoid enhancer-binding factor 1), and ZIC3.^28,29^ FOXF2 is expressed by ECs^28,29^ and pericytes^30^ and is critical to maintain BBB TJ integrity and low transcytosis.^30^ ZIC3 is upregulated during development, suggesting a potential role in BBB establishment.^29,31^ Overexpression of FOXF2 alone in iPSC-derived ECs^32^ upregulates OCLN (occludin) mRNA expression and Wnt (wingless-related integration site)/β-catenin signaling,^33^ a pathway critical for BBB development (reviewed in the study by Engelhardt and Liebner^34^). Moreover, overexpression of FOXF2 and ZIC3 in primary human umbilical vein ECs upregulates a limited set of BBB-specific genes.^28^ These suggest that FOXF2 and ZIC3 may reprogram iPSC-derived generic ECs toward a BBB (ie, brain) molecular and phenotypic identity.

Here, we describe a robust and reproducible method to generate iPSC-derived reprogrammed BMECs (rBMECs) by combining an iPSC differentiation step into BBB-primed ECs (bpECs)^35^ with FOXF2- and ZIC3-mediated transcriptional reprogramming. rBMECs show higher levels of key BBB transcripts and proteins compared with primary HBMECs and bpECs, consistently exhibit higher TEER, lower transcellular transport, and equivalent efflux transport compared with HBMECs at the functional level, and respond to angiogenic stimuli. rBMECs interact with iPSC-derived astrocytes and pericytes to form an NVU when they are cocultured in a 3D microfluidic MIMETAS system with flow, acquire high TEER levels, and exhibit low barrier permeability to tracers, resembling at some level the in vivo human NVU/BBB. Finally, we find that rBMECs expressing the familial Alzheimer disease (fAD) mutation *APP V717I* show decreased barrier integrity and upregulation of inflammatory markers. In contrast, treatment of control rBMECs with oAβ42 (oligomeric amyloid-β [1-42] peptide) increases inflammatory markers but does not impair barrier integrity. Taken together, rBMECs enable a more faithful in vitro modeling of the human BBB compared with prior studies, thus enabling future studies of disease pathogenesis and development of putative therapeutics. Moreover, the *APP V717I* mutation found in fAD alters several BBB-related properties of rBMECs and their inflammatory state, likely independent of Aβ42 (amyloid-β [1-42] peptide) production.

## Methods

### Data Availability

All RNA sequencing data generated by this study have been made publicly available at the Gene Expression Omnibus repository and can be accessed with the reference number GSE267498. All other data that support the findings of this study are available from the corresponding author upon reasonable request.

### hiPSC Line Maintenance

All experiments were performed using the human control iPSC line IMR90-cl.4 (WiCell).^36^ IMR90-cl.4 iPSCs with heterogenous or homozygous *APP*^*Lon*^ (*V717I*) mutation^37,38^ were also used for experiments described in Figure 5, and the mutation was confirmed by genotyping before differentiation. For key validation of EC identity and BBB structural and functional properties, we repeated a subset of experiments in other iPSC lines, namely, human control iPSC line FA0000010 (FA10), ND50031 (NCRM-5), and ND50025 (NCRM-4; RUCDR). IMR90-cl.4 and NCRM-4 were derived from female donors, while FA10 and NCRM-5 were from male donors. All commercial cell lines were previously authenticated and tested for pluripotency.^36,39^ For differentiation into bpECs or neural progenitor cells (NPCs), iPSCs were maintained on feeder culture of irradiated CF1 mouse embryonic fibroblasts with the human embryonic stem cell medium: knockout DMEM (Dulbecco’s Modified Eagle Medium), 20% knockout serum replacement (Thermo Fisher), 2-mmol/L Glutamax (Thermo Fisher), 0.1-mmol/L MEM nonessential amino acids (Thermo Fisher), 1% penicillin-streptomycin (Thermo Fisher), and 0.1-mmol/L β-mercaptoethanol (Thermo Fisher) supplemented with 20-ng/mL bFGF (basic fibroblast growth factor; R&D) and passaged when 80% to 90% confluent. Feeder-free hiPSCs were maintained in StemFlex medium (Thermo Fisher) containing penicillin-streptomycin (Thermo Fisher) on Cultrex-coated plates (Bio-Techne). hiPSCs were frozen in 45% knockout serum replacement, 45% StemFlex, and 10% dimethyl sulfoxide. Cultures were passaged 1:3-1:6 using ReLeSR (StemCell Technologies) every 3 to 4 days. Mycoplasma testing was performed when cells were initially acquired and periodically thereafter. Please see the Major Resources Table in the Supplemental Material for more details.

**Figure 1.**
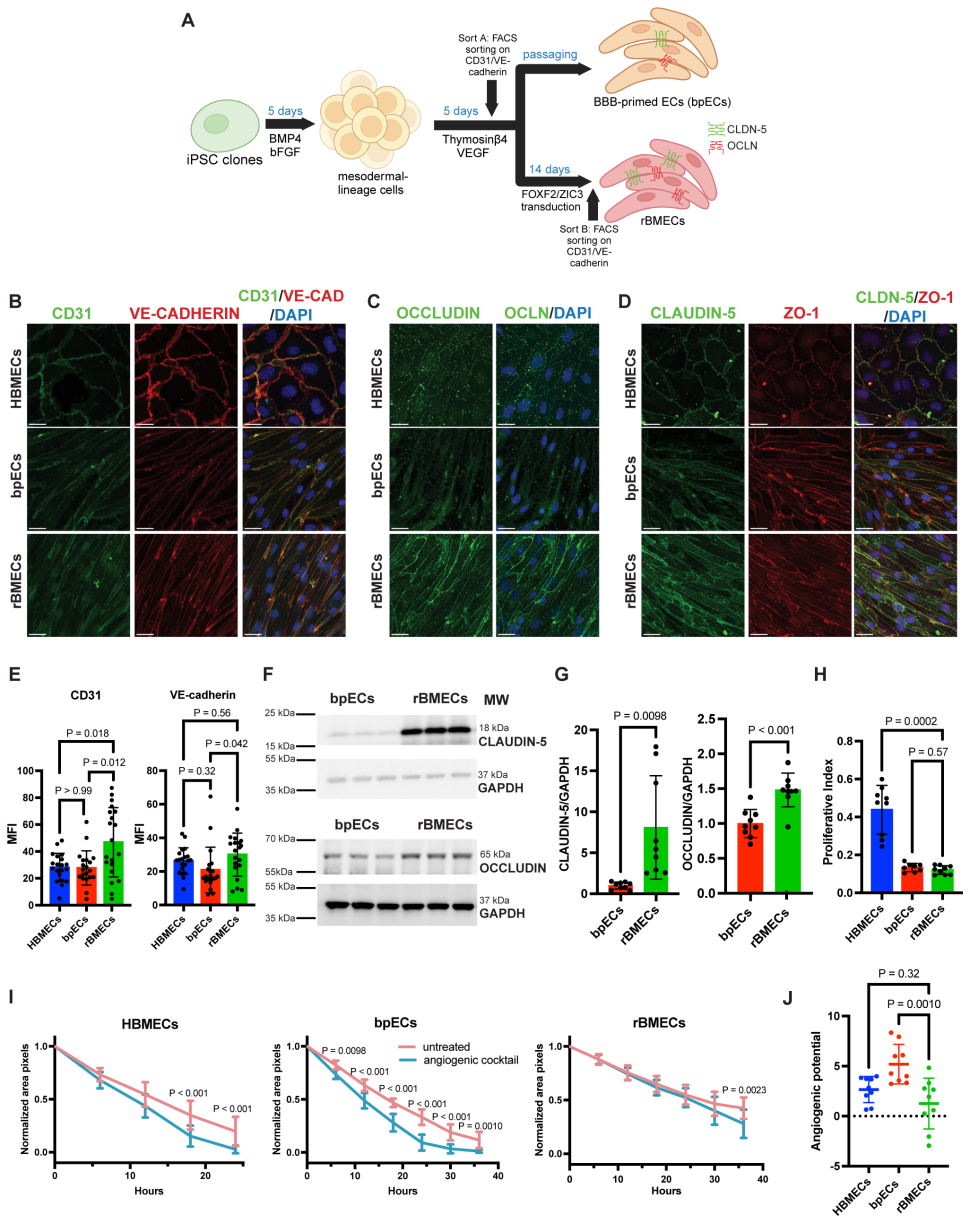
**Reprogrammed brain microvascular endothelial cells (rBMECs) generated through **FOXF2** (forkhead box F2)/ZIC3 (zic family zinc finger 3) transcriptional reprogramming express key endothelial cell (EC) markers and respond to angiogenic stimuli. A**, Schematic for the generation of blood-brain barrier (BBB)–primed ECs (bpECs) and rBMECs from human induced pluripotent stem cells (hiPSCs). Created in BioRender.com. **B** through **D**, Representative immunofluorescence staining of human brain microvascular ECs (HBMECs), IMR90 hiPSC–derived bpECs and rBMECs for EC (CD31 and VE-cadherin [vascular endothelial cadherin]) and TJ (tight junction; OCLN [occludin], CLDN-5 [claudin-5], and ZO-1 [zonula occludens-1]) proteins, and 4′,6-diamidino-2-phenylindole (DAPI; blue). Scale bar, 25 µm. **E**, Quantification of CD31 and VE-cadherin mean fluorescence intensity (MFI) from immunofluorescence staining images, normalized to total DAPI^+^ cells in each image; n=20 from 4 independent differentiations, Welch’s ANOVA (CD31) and 1-way ANOVA (VE-CAD). **F** and **G**, Representative Western blotting (WB) and quantification for CLDN-5 and OCLN expression. Mean±SD, n=9 replicates from 3 independent differentiations, Welch’s *t* test (CLDN-5) and unpaired *t* test (OCLN). **H**, Proliferative index quantification after cell migration assay, n=8 to 9 replicates from 3 independent differentiations, Welch’s ANOVA. **I**, Quantification of the normalized area (pixels) of the wound region in the scratch assay, n=9 replicates from 3 independent differentiations, repeated measures 2-way ANOVA. **J**, Quantification of angiogenic potential, n=9 replicates from 3 independent differentiations, 1-way ANOVA.

**Figure 2.**
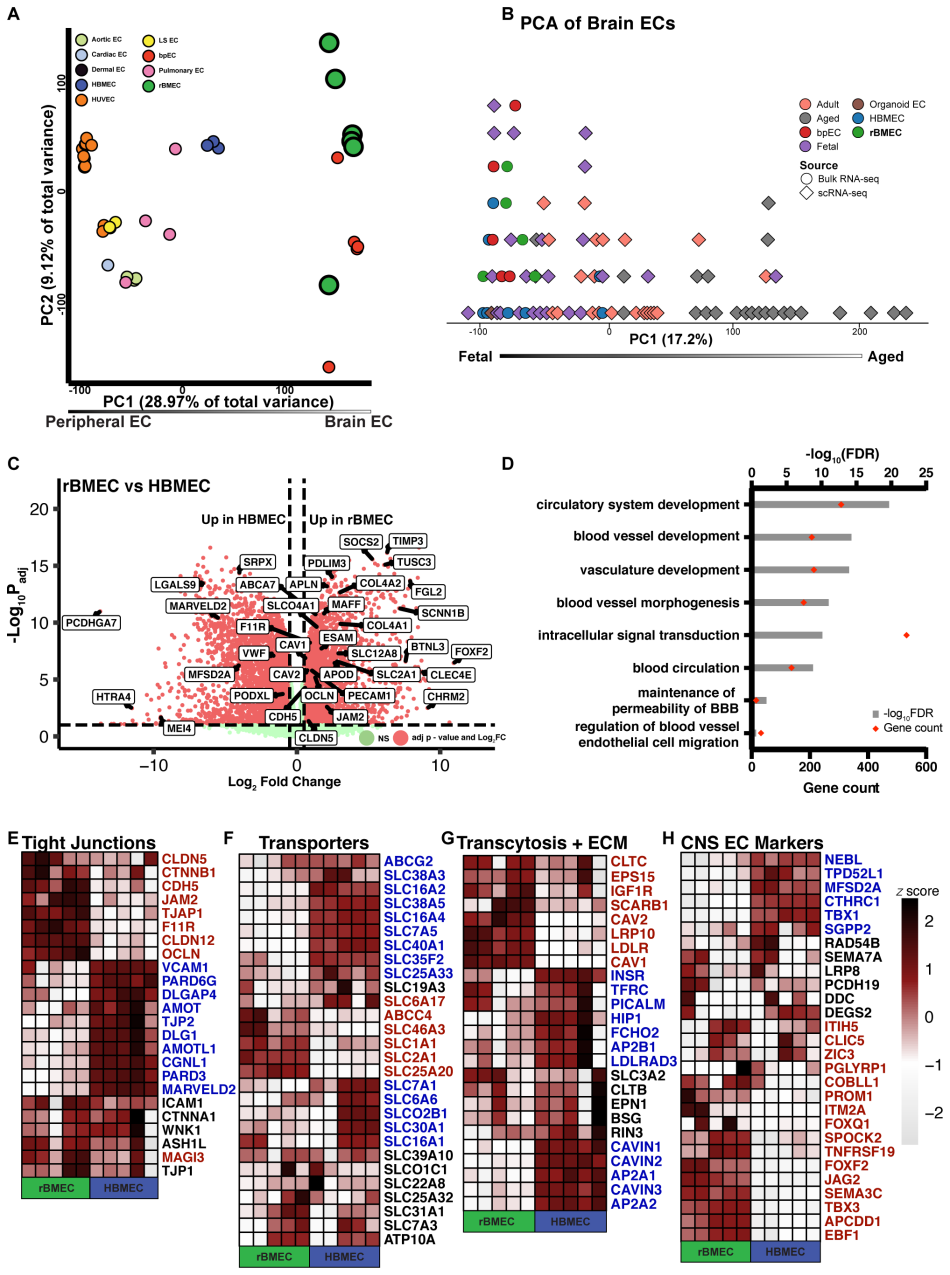
**The reprogrammed brain microvascular endothelial cell (rBMEC) transcriptome shows higher expression of brain endothelial cell (EC) identity genes and the blood-brain barrier (BBB) transcriptome compared with primary human brain microvascular ECs (HBMECs). A**, Principal component analysis (PCA) plot illustrating peripheral vs brain EC identity of 32 differentiated and primary EC samples from published studies and this study (see Methods section for more details). **B**, PCA plot illustrating fetal vs adult or aged brain EC identity characteristics of 80 EC RNA samples from published studies and this study. **C**, Volcano plot highlighting upregulated and downregulated BBB-specific genes in IMR90 human induced pluripotent stem cell (hiPSC)–derived rBMECs compared with HBMECs. All listed BBB-specific genes are significant (*P*_adj_<0.05; |logFC|>0.25). **D**, Upregulated gene ontology (GO) analysis pathways in rBMECs compared with HBMECs. Red dots label gene counts, while gray bars indicate −log(false discovery rate) values. **E** through **H**, Heatmap of BBB-specific genes (TJ [tight junction] proteins, transporters, receptor-mediated transcytosis, extracellular matrix, and brain EC identity genes) between rBMEC and HBMECs. The scale bar is log(*Z* score), where dark colors indicate a high *Z* score and light colors indicate a low *Z* score. Genes upregulated in rBMECs are labeled in red text, and genes upregulated in HBMECs are labeled in blue text. CNS indicates central nervous system.

**Figure 3.**
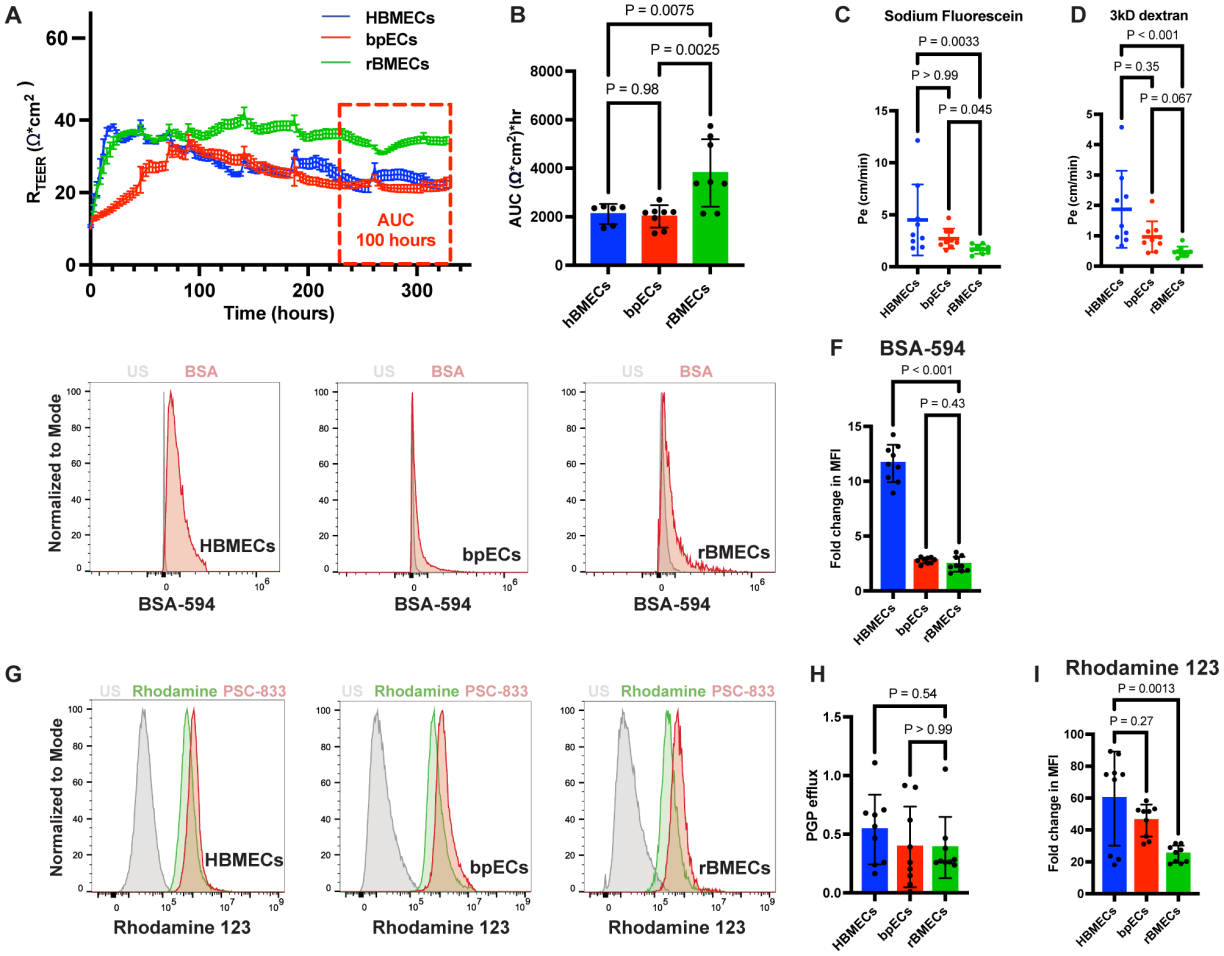
**Reprogrammed brain microvascular endothelial cells (rBMECs) have strong functional blood-brain barrier (BBB) properties and respond to inflammatory stimuli. A** and **B**, Representative transendothelial electrical resistance (TEER) measurements obtained with an electric cell-substrate impedance sensing (ECIS) instrument over 320 hours and dotted bar graph of the area under the curve (AUC) quantification for IMR90 human induced pluripotent stem cell (hiPSC)–derived BBB-primed ECs (bpECs), rBMECs, and primary human brain microvascular endothelial cells (HBMECs). The red box indicates the period used for the AUC quantification. Mean±SD, n=6 to 8 replicates from 3 to 4 independent differentiations, 1-way ANOVA. **C** and **D**, Dotted bar graphs of the transwell permeability across endothelial cell (EC) monolayers to sodium fluorescein (NaF) and 3-kDa dextran. Mean±SD, n=9 replicates from 3 independent differentiations, the Kruskal-Wallis test. **E** and **F**, Representative histograms and quantification of BSA-AF594 (bovine serum [BSA], alexa fluor 594 conjugate) uptake in HBMECs, bpECs, and rBMECs. Mean±SD, n=9 replicates from 3 independent differentiations, Welch’s ANOVA. **G** through **I**, Representative histograms of Rhodamine 123 median fluorescence intensity (MFI) and PGP (P-glycoprotein) efflux in HBMECs, IMR90 hiPSC–derived bpECs, and rBMECs. n=9 replicates from 3 independent differentiations, 1-way ANOVA.

**Figure 4.**
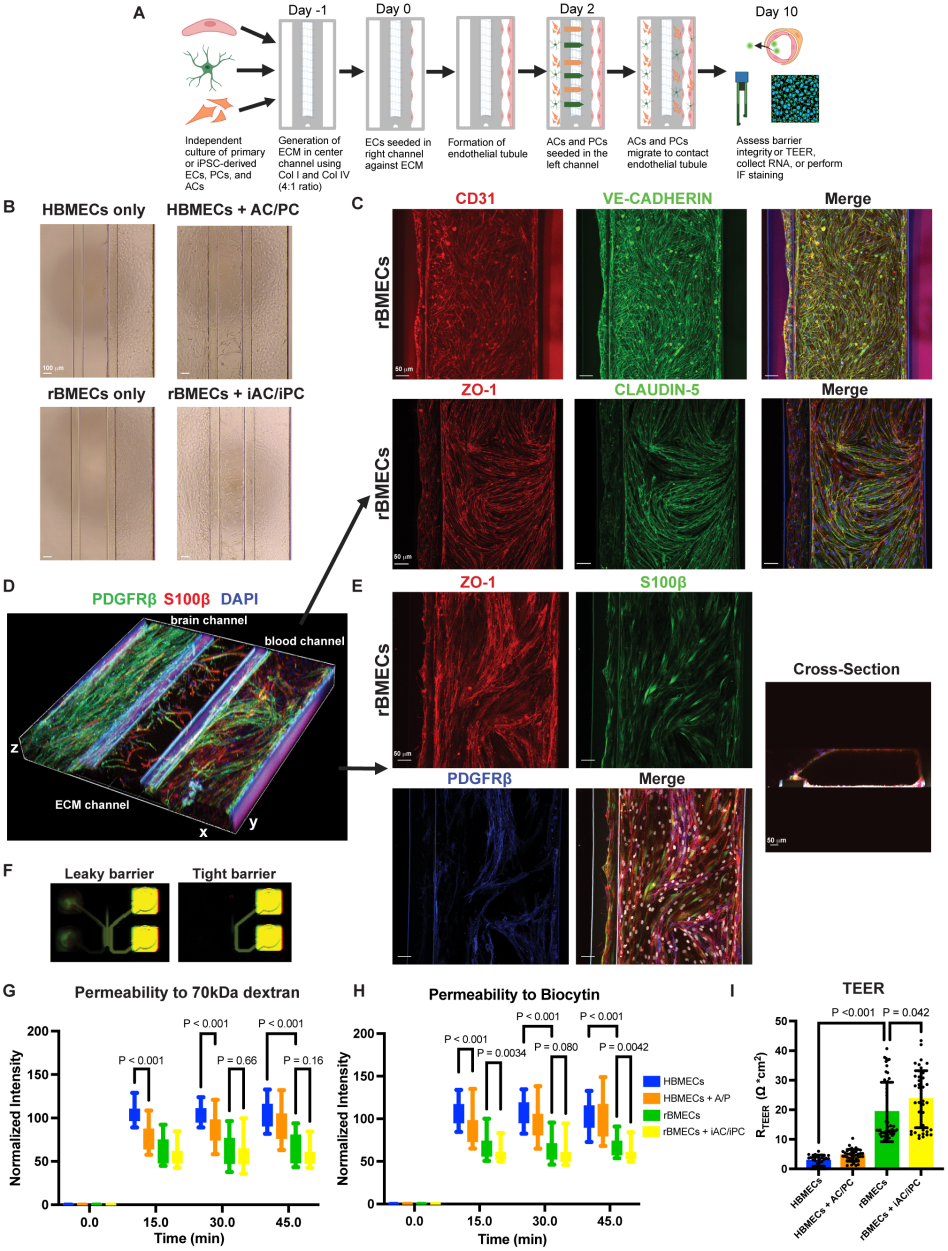
**Reprogrammed brain microvascular endothelial cell (rBMEC) tubules have improved barrier properties when cocultured with induced pluripotent stem cell (iPSC)–derived astrocytes (ACs) and pericytes (PCs) in a 3D microfluidic neurovascular unit (NVU) system. A**, Schematic diagram for generating the 3D NVU/blood-brain barrier (BBB) microfluidic system using the MIMETAS platform. Created in BioRender.com. **B**, Brightfield images of endothelial cell (EC) tubules formed by human brain microvascular ECs (HBMECs) and rBMECs without (**left**) and with (**right**) primary or iPSC-derived ACs and PCs, respectively. **C**, Representative immunofluorescence (IF) staining of rBMEC tubules for CD31 (red) and VE-cadherin (vascular endothelial cadherin; green, top) or ZO-1 (zonula occludens-1; red) and CLDN-5 (claudin-5; green, bottom). Scale bar, 50 µm. **D**, 3D image of IF staining within the 3D NVU microfluidics MIMETAS system showing iPSC-derived PCs (iPCs; PDGFRβ [platelet-derived growth factor receptor, beta]) and iPSC-derived astrocyte (iACs; S100β [S100 calcium-binding protein β]) migrating from the brain channel across the ECM channel to the blood channel where the rBMEC tubules are located. **E**, Representative triculture IF staining of rBMECs (ZO-1, red), iACs (S100β, green), and iPCs (PDGFRβ, blue) in the blood channel; cross-sectional view shows an rBMEC tubule with a lumen. **F**, Representative image showing diffusion of tracers through leaky and tight EC 3D tubules in the MIMETAS microfluidics system. **G** and **H**, Box plots show the normalized intensity of 70-kDa dextran and biocytin (890-Da) tracers in the middle channel, indicative of tracer permeability across either the rBMEC or HBMEC tubules; n=36 to 41 NVU chips from 3 independent differentiations, 2-way ANOVA. **I**, Dotted bar graph of the transendothelial electrical resistance (TEER) measurement in the 3D NVU/BBB microphysiological MIMETAS system using the OrganoTEER instrument for HBMEC or rBMEC tubules alone or together with primary PCs/ACs or iPSC-derived iPCs/iACs; n=30 to 48 chips over 3 independent differentiations, Welch’s ANOVA.

**Figure 5.**
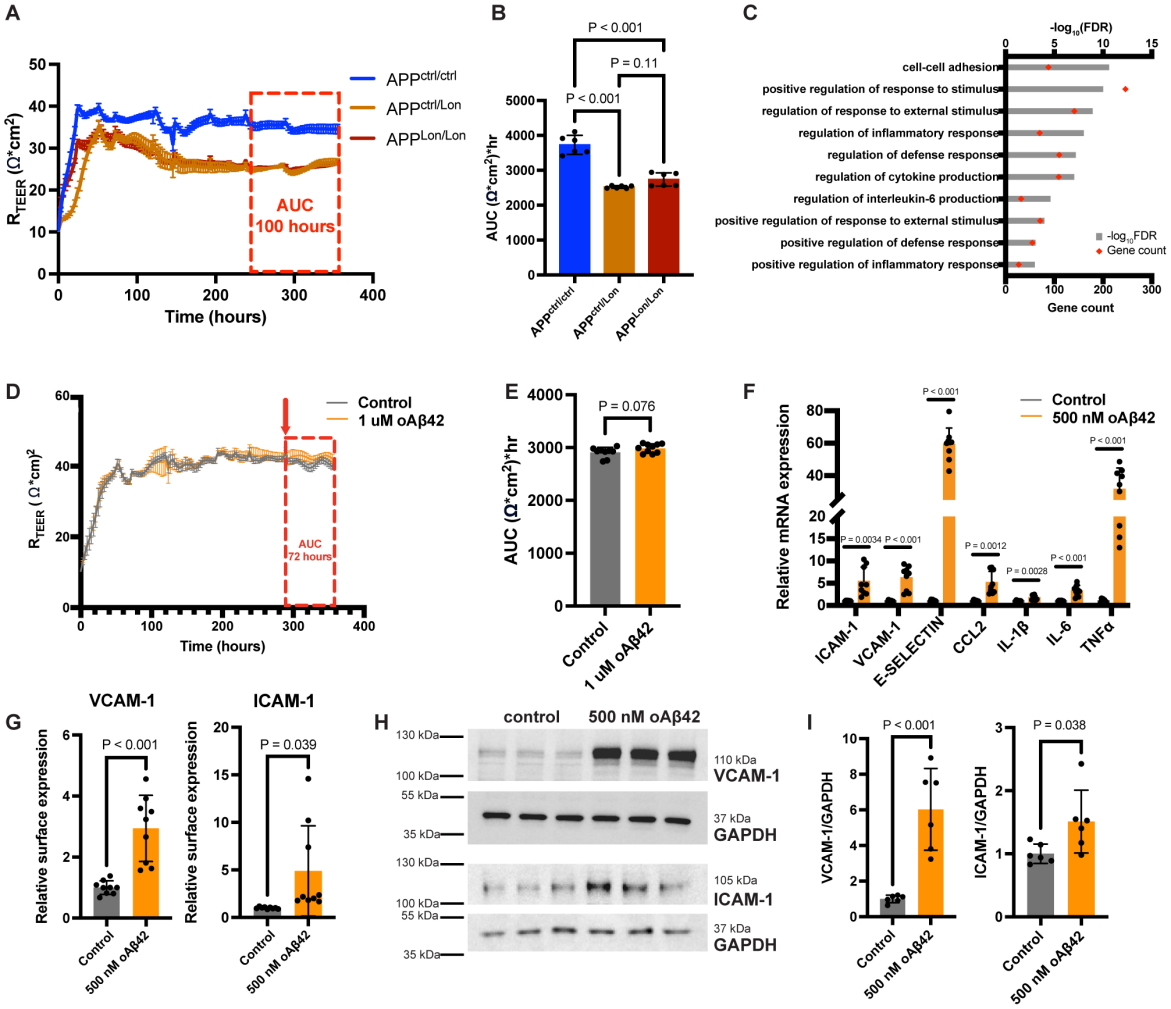
**Reprogrammed brain microvascular endothelial cells (rBMECs) can be used to study neuroinflammation and barrier dysfunction in Alzheimer disease. A** and **B**, Representative transendothelial electrical resistance (TEER) measurement for isogenic *APP*^*ctrl/ctrl*^, *APP*^*ctrl/Lon*^, and *APP*^*Lon/Lon*^ rBMECs and dotted bar graph for area under the curve (AUC) quantification (red box). Mean±SD, n=6 from 2 independent differentiations, Welch’s ANOVA. **C**, Gene ontology analysis showing upregulated pathways in *APP*^*Lon*^ rBMECs compared with control rBMECs. Red dots label gene counts, while gray bars indicate −log(false discovery rate) values. **D** and **E**, Representative TEER measurement for rBMECs during 72-hour treatment with F12 vehicle or 1-µM oAβ42 (oligomeric amyloid-β [1-42] peptide; red box), dotted bar graph for AUC quantification, n=9 to 10 replicates from 3 differentiations, unpaired *t* test. **F**, RT-qPCR (reverse transcription-quantitative polymerase chain reaction) for several inflammatory genes in IMR90 human induced pluripotent stem cell (hiPSC)–derived rBMECs after 6 hours of 500-nM oAβ42 treatment; n=8 to 9 replicates from 3 independent differentiations, Welch’s *t* test. **G**, VCAM-1 (vascular cell adhesion molecule-1) and ICAM-1 (intercellular adhesion molecule-1) surface expression quantification after 6 hours of 500-nM oAβ42; n=6 replicates from 2 independent differentiations, the Welch *t* test. **H** and **I**, Representative Western blots and quantification for VCAM-1 and ICAM-1 after 6 hours of 500-nM oAβ42; n=6 replicates from 2 independent differentiations, unpaired *t* test.

### Directed Differentiation of hiPSCs Into bpECs

hiPSCs were differentiated into bpECs as previously reported with slight modifications.^35^ hiPSCs were dissociated with collagenase-IV, and cell aggregates were plated in chemically defined media: 50% IMDM (Thermo Fisher), 50% Ham’s F12 (Thermo Fisher), 5-mg/mL BSA (Thermo Fisher), 15-µg/mL transferrin (Sigma-Aldrich), 0.1-mmol/L β-mercaptoethanol (Thermo Fisher), 7-µg/mL insulin (Sigma-Aldrich), 1% penicillin-streptomycin (Thermo Fisher), or 1-µg/mL fibronectin (Sigma-Aldrich) coated 10-cm dishes from one well of a 6-well plate to 1 10-cm dish. Chemically defined media were supplemented with 10-ng/mL BMP4 (R&D) for 1.5 days and 50-ng/mL BMP4 and 20-ng/mL bFGF (R&D) for the next 3.5 days for the generation of mesodermal lineage cells. From days 5 to 10, endothelial differentiation was initiated by supplementing chemically defined media with 50-ng/mL VEGF (vascular endothelial growth factor)-A_165_ (R&D) and 100-ng/mL thymosin-β4. ECs were purified at days 10 and 11 by fluorescence-activated cell sorter (FACS) for CD31^+^/cadherin-5^+^ cells. These ECs, now termed bpECs, were plated on collagen IV/fibronectin-coated plates at a density of 30 000 to 35 000 cells/cm^2^ and fed EC medium (human endothelial serum-free medium [Thermo Fisher]) supplemented with 1% human serum (Sigma-Aldrich) supplemented with 50-ng/mL VEGF-A_165_ (R&D), 20-ng/mL bFGF (R&D), and 10-μM SB431542 (Sigma-Aldrich). bpECs were expanded and passaged 1:2 to 1:3 every 7 to 10 days and generally used for 5 passages.

### Reprogramming of bpECs Into rBMECs

bpECs were plated at 30 000 to 35 000 cells/cm^2^ on collagen IV/fibronectin-coated plates using EC media supplemented with 50-ng/mL VEGF165, 20-ng/mL bFGF, and 10-µM SB431542. On day 2, bpECs were transduced with FOXF2 (≈1×10^6^ lentiviral p24 units/mL of medium) and ZIC3 (≈7.5×10^5^ lentiviral p24 units/mL of medium) as determined by Lenti-X GoStix (Takara). Successfully infected cells were selected with 1-µg/mL puromycin (Sigma) for 24 to 48 hours. Infected bpECs were purified via FACS for CD31^+^/VE-cadherin (vascular endothelial cadherin)^+^ cells on day 14. FACS-purified and infected bpECs, referred to as rBMECs, were plated on collagen IV/fibronectin-coated plates at 25 000 to 35 000 cells/cm^2^ and expanded until confluent. rBMECs were passaged at high density using 1:2 to 1:3 splits every 7 to 10 days and used within 5 passages.

### Generation of Lentiviral Vectors and Lentiviruses

FOXF2 and ZIC3 were subcloned into pLex_307, which was a gift from David Root (Addgene), and verified by Sanger sequencing (Azenta). FUW-tetO-NFIA was a gift from Lorenz Studer (Addgene).^40^ FUdeltaGW-rtTA was a gift from Konrad Hochedlinger (Addgene).^41^ psPAX2 was a gift from Didier Trono (Addgene). VSV.G was a gift from Tannishtha Reya (Addgene).^42^ Lentiviral particles for FOXF2, ZIC3, NFIA (nuclear factor 1 A-type), and rtTA (reverse tetracycline-controlled transactivator) were generated in HEK293 as described previously,^43^ using the psPAX2/VSV.G second-generation system. Efficacy of lentiviral particles was confirmed by infection and subsequent qPCR (quantitative polymerase chain reaction).

### Fluorescence-Activated Cell Sorter

Cells were dissociated into a single-cell suspension using Accutase (Thermo Fisher) and washed with basal medium. Cell suspensions were incubated with FACS buffer (10% knockout serum replacement in DPBS) with fluorescently tagged antibodies (CD31-fluorescein isothiocyanate and VE-cadherin-APC) for 30 minutes at room temperature, protected from light. Cell suspensions were washed with FACS buffer, and cell pellets were resuspended for FACS by filtering through 0.45-μM filters (Fisher Scientific) to remove cell aggregates. bpECs or rBMECs were sorted on the Sony MA900 cell sorter, and CD31^+^/VE-cadherin^+^ cells were subsequently expanded.

### Primary EC, Pericyte, and Astrocyte Cultures

HBMECs (Cell Systems) were cultured in Endothelial Cell Growth Medium MV 2 (Promocell). Pericytes were cultured in pericyte media (PM; ScienCell), and astrocytes were cultured in astrocyte media (AM; ScienCell). HBMECs and pericytes were plated onto collagen IV and fibronectin-coated plates, while astrocytes were plated onto either Matrigel- or Cultrex-coated plates. All cells were supplemented with media every other day and passaged using Accutase once 90% confluent.

### Generation of iPSC-Derived Pericytes

Briefly, feeder-free iPSCs were differentiated into neural crest pericytes following an established protocol, where iPSCs were treated with a neural crest differentiation medium supplemented with CHIR99021 and subsequently treated with PM (ScienCell).^44^ Cells were later sorted on CD146+/PDGFRβ+ to ensure a homogeneous population before introduction into the 3D NVU triculture system.

### Generation of iPSC-Derived Astrocytes

hiPSCs were first differentiated into NPCs using dual-SMAD inhibition in embryoid bodies as previously described^45^ and then differentiated into astrocytes (iPSC-derived astrocytes [iACs]).^46^ On day −1, NPCs were dissociated using Accutase and plated at a density of 15 000 cells/cm^2^ in N2/B27 media on Cultrex-coated plates. On day 0, the media was replaced with supplemented AM (ScienCell). On day 1, differentiating cells were transfected with lentiviral particles carrying dox-inducible NFIA and rtTA (Addgene). Cells were fed every other day using AM supplemented with doxycycline (2 μg/mL; Sigma-Aldrich). Cells were split at 90% to 95% confluence roughly once every 7 days and replated at a density of 15 000 cells/cm^2^ for 30 days. At this stage, astrocytes were split 1:3 once 90% to 95% were confluent and were expanded, banked, and utilized.

### RNA Isolation and RT-qPCR

RNA was extracted using the RNeasy Plus Micro Kit (Qiagen) and resuspended in 30 µL of RNAse-free water. RNA quantity and quality were assessed with a Nanodrop One (Thermo Scientific). For qPCR, 100 to 250 ng of RNA was transcribed into cDNA using the Maxima First Strand cDNA Synthesis Kit (Thermo Fisher). Gene expression was assessed using the FAST SYBR Green Master Mix (Thermo Fisher Scientific) with primer sets (Integrated DNA Technologies) for target genes on a QuantStudio 7 Flex system (Applied Biosystems) and CFX Opus 384 (Bio-Rad). Gene expressions for target genes were normalized to GAPDH, and fold changes were calculated as ΔΔC_t_.

### Western Blotting

ECs were harvested using RIPA Lysis Buffer supplemented with Halt protease and phosphatase inhibitors single-use cocktails (Thermo Fisher) on ice; 10 μg of lysates and 1× Laemmli sample buffer/5% β-mercaptoethanol were boiled at 95° C for 5 minutes. SDS-PAGE was performed by loading samples on Bolt 4% to 12% gradient Bis-Tris gels using Bolt MOPS buffer (Thermo Fisher) or Tris-Acetate 3% to 8% gels using NuPAGE Tris-Acetate Running Buffer for ABCC4 (Thermo Fisher). Nitrocellulose membranes were first blocked with Superblock TBS buffer/0.1% Tween-20 (Thermo Fisher) for 1 hour and then incubated overnight at 4° C with the following primary antibodies diluted in Superblock/0.1% Tween-20: CLDN-5 (claudin-5; 1:1000; Thermo Fisher), OCLN (1:1000; Thermo Fisher), VCAM-1 (vascular cell adhesion molecule-1; 1:1000, Abcam), pSTAT3 (phosphorylated signal transducer and activator of transcription-3; 1:1000, BioLegend), STAT3 (signal transducer and activator of transcription 3; 1:1000, BioLegend), total CAV1 (caveolin-1; 1:1000, Sigma-Aldrich), p-CAV1 (phosphorylated caveolin-1; 1:1000, Cell Signaling), ABCC4 (ATP-binding cassette sub-family C member 4; 1:1000, Cell Signaling), and GAPDH (1:5000; Sigma). After 3 washes with TBS/0.1% Tween-20, membranes were then incubated with the appropriate HRP-conjugated secondary antibody (1:5000, Thermo Fisher) or IRdye 800 secondary antibody (1:20 000, LicorBio) for 1 hour and washed 3 more times with TBS/0.1% Tween-20. Pierce ECL Western blotting substrate or SuperSignal West Pico PLUS chemiluminescent substrate (Thermo Fisher) was used to develop chemiluminescence, and blots were imaged using the Bio-Rad Chemidoc MP system. IRDye-stained blots were imaged directly using the 800 Channel on the LICOR Odyssey SA.

### TEER Measurement

TEER measurements were performed using the ECIS (Electric Cell-substrate Impedance Sensing) Z-theta instrument on the 96W20idf array at 4000-Hz frequency. Briefly, 20 000 cells/well were plated on poly-D-lysine and collagen IV/fibronectin-coated 96-well electrode array plates in the respective medium for each cell type. Resistance was recorded for 250 to 300 hours, and the medium was changed every other day. The resistance curve was generated using PRISM (GraphPad), and the area under the curve was measured either for the last 100 hours of recording or for the duration of oAβ42 or cytokine treatment (72 or 24 hours) from 6 to 8 wells/sample using the area under the curve PRISM function.

### Tracer Permeability Assay

Tracer permeability assay for the determination of paracellular permeability was performed as previously described.^47^ rBMECs, bpECs, and hBMECs were cultured on 12-well polycarbonate Transwell inserts (0.4 µM, Corning) for 10 days to ensure a complete monolayer. Growth factors were removed for 24 hours before assay. A full media change was performed on the bottom chamber (1.5 mL), and all 3 tracers (sodium fluorescein, 10 µg/mL; 3-kDa dextran, 25 µg/mL; and 70-kDa dextran, 25 µg/mL) were added to the top chamber in 0.5-mL medium to start the assay. As soon as tracers were added, 100-µL medium was collected from the bottom chamber as a 0-minute collection and replaced with 100-µL fresh media for each well, followed by serial collection every 10 minutes for up to 60 minutes. Fluorescent intensities were measured, and clearance slopes were calculated using

ΔV=C_bottom_×V_bottom_/C _upper._

C_bottom_ and C_upper_ are fluorescent intensities measured for each tracer in the top and bottom chambers, respectively, and V_bottom_ is the volume of media in the bottom chamber (1.5 mL)

P_filter_=PS_filter_/s and P_cell+filter_=PS_cell+filter_/s.

P_filter_ and P_cell+filter_ are permeability coefficients for blank insert and insert with cells, and PS_filter_ and PS_cell+filter_ can be obtained by a regression plot of ΔV against time for blank insert and insert with cells

1/P_e_=1/P_cell+filter_−P_filter_.

Pe is the permeability coefficient for EC monolayers.

### Wound Scratch Assay

Cells were plated at a density of 100K/well (24-well plate). Once confluent, cells were fed their standard media absent VEGF and bFGF supplementation. Monolayers were scratched in the center of each well using the Autoscratch machine (BioTek). Wells were then treated with either control media, 100-ng/mL VEGF, or an angiogenic cocktail consisting of VEGF (37.5 ng/mL), bFGF (37.5 ng/mL), and HGF (37.5 ng/mL). Plates were cultured and imaged automatically every 6 hours using the Cytation5 plate reader (BioTek) and software for HBMECs (24 hours) and bpECs and rBMECs (36 hours) during culture in a BioSpa incubator (BioTek). Each well was quantified for the % surface area of the wound utilizing an ImageJ plug-in for identifying the area of the scratch,^48^ which was then normalized to the original size of the wound for each well. To compare cell types, we calculated an angiogenic potential, where


angiogenicpotential=∫untreatedwoundarea−∫woundareawithangiogeniccocktail.

### Ki67 Quantification Assay

After the wound scratch assay was performed, cells were fixed with 4% PFA. Cells were blocked as described above and stained with Ki67 (Antigen Kiel 67) antibody (1:500). Cells were labeled with a GFP (green fluorescent protein) secondary antibody and 4′,6-diamidino-2-phenylindole (DAPI). Wells were imaged for DAPI and GFP fluorescence on the BioSpa as done previously. Each well was quantified for proliferative index, where


$$ProliferativeIndex=\frac{NumberofKi67+cells}{NumberofDAPI+cells}.$$

Cells were identified as Ki67 or DAPI positive using the annotation machine learning tool from the QuPath software.^49^

### Uptake and Transporter Assays

As previously described,^50^ cells were plated at a density of 100K/well (24-well plate) and subcultured until confluent. First, cells were incubated with either 1-mg/mL unlabeled BSA or 1-mg/mL BSA-Alexa 594 for 1 hour at 37 °C. Cells were then dissociated into a single-cell suspension, stained for eFluor 780, fixed with 4% PFA, and analyzed for Alexa594 median fluorescence intensity (MFI). For the transporter assay, cells were incubated with Rhodamine 123 (10 µM) for 1 hour at 37 °C. To assess the effect of PGP (P-glycoprotein) efflux, cells were pretreated with PSC-833 (a specific PGP inhibitor) for 1 hour before Rhodamine treatment. Cells were dissociated into a single-cell suspension, stained for eFluor 780, fixed with 4% PFA, and analyzed for Rhodamine MFI. NPCs were utilized as a positive control due to their low level of PGP efflux. Data were normalized to the unstained MFI for each cell type, which we termed fold change in MFI. To calculate PGP efflux, we calculated the percent change in MFI with and without PSC-833


$$PGPef\;flux=\frac{MFI_{PSC\text{\textasciitilde{}}\text{-}833}-MFI_{Rhodamine}}{MFI_{Rhodamine}}.$$

### Amyloid-β42 Oligomerization and Treatment

Amyloid-β42 was monomerized, stored, and later oligomerized as previously described.^51,52^ In brief, Aβ(1-42; rPeptide, 1 mg) vial was allowed to equilibrate to room temperature. Peptides were resuspended in ice-cold hexafluoroisopropanol (Sigma-Aldrich) to achieve a 1-mmol/L concentration directly in a vacuum-sealed vial using a Hamilton syringe. The suspension was divided into 8 equal aliquots by volume using a Hamilton syringe into low protein–binding Eppendorf tubes, incubated at room temperature for 2 hours, and dried on speedvac at 800*g* for 25 °C. Upon drying, monomerized Aβ(1-42) films can be stored at −80 °C in sealed tubes. For generating oligomers, Eppendorf tubes containing Aβ(1-42) peptide films were equilibrated to room temperature, resuspended in dimethyl sulfoxide to achieve a 5-mmol/L concentration, and sonicated at 17 °C for 10 minutes for complete resuspension. Oligomerization was induced by adding phenol red–free F12 to achieve a final concentration of 100 µM and incubating at 4 °C for 24 to 48 hours. rBMEC monolayers were treated with oAβ at 500 nmol/L or 1 µM for the indicated time, and F12 was used as a vehicle control.

### 3D Microfluidic NVU Culture Model

OrganoPlate 3-lane-64 plates (MIMETAS) were used to generate a 3D microfluidic system of the NVU. In each chip, the center channel was filled with an ECM gel, an endothelial tubule was generated in the right channel, and a mixture of iPSC-derived astrocytes and pericytes was seeded in the left channel and allowed to migrate toward the ECs during culture upon a bidirectional OrganoFlow rocker (MIMETAS).

#### ECM Generation and Endothelial Channel Coating

On day −1, the center ECM channel was filled with a 4:1 ratio of collagen I (5 mg/mL) and collagen IV solution (5 mg/mL), incubated at 37 °C for 15 min, and covered with PBS to prevent the collagen gel from drying. The right channel inlet was coated using fibronectin (10 µg/mL) and collagen IV (10 µg/mL) overnight.

#### EC Seeding

On day 0, ECs were singularized and resuspended to achieve a 15 000-cells/µL concentration. Cells were seeded in the right channel outlet at a density of 30 000 cells/chip. The plate was placed on a 75º plate stand from the manufacturer and incubated at 37 °C for 4 to 5 hours to allow ECs to settle against the gel. Then, outlets were replenished with endothelial media, and the plate was placed on the OrganoFlow rocker to alternate between −7 and 7^o^ every 8 minutes. After 1–2 days, ECs formed an open tubule in the right channel.

#### Astrocyte and Pericyte Channel Coating and Cell Seeding

On day 1, the left channel inlet was coated with Matrigel. Primary astrocytes and iPSC-derived astrocytes were singularized and resuspended in AM-PM media (2:1 ratio of AM:PM) to achieve a 15 000-cells/µL concentration. Primary pericytes and iPCs were similarly singularized and resuspended in AM-PM to achieve a 7500-cells/µL concentration. Primary astrocytes and primary pericytes were mixed at a ratio of 1:1 and seeded in the left channel outlet for each chip to achieve 15 000 astrocytes and 7500 pericytes per chip. The plate was rested on the stand for 4 to 5 hours at 37 °C to allow pericytes and astrocytes to settle against the gel. Finally, outlets were replenished with AM-PM, and the plate was placed back on the OrganoFlow rocker for continuous culture until day 10. Media was replenished every other day. Beginning on day 6, chips were replenished with their standard EC media without bFGF and VEGF-A supplementation.

### Tracer Permeability Assay

On day 10 post-seeding, a 70-kDa dextran (0.5 µg/mL) and biocytin tracer (0.5 µg/mL) were introduced into the right inlet and outlet wells (40 and 30 µL, respectively). The gel inlet, left inlet, and left outlet wells were filled with 20-µL media without tracer. Then, the plate was placed onto the OrganoFlow rocker and imaged every 15 minutes in the LI-COR. The fluorescence intensity for each well was quantified with the Odyssey SA system. Analysis was performed as the normalized fluorescence intensity over the ECM channel center well, which represented the leakage from the right (vascular) channel into the left (brain) channel


$$NormalizedIntensity=\frac{MiddleECMchannelintensity_{sample}}{Meanmiddlechannelintensity_{ECM}}.$$

where ECM chips only had a control gel, representing the leakage without cells present.

### 3D TEER Assay

Chips were assayed for TEER on day 10 post-seeding. After the plate was introduced into the OrganoTEER device, TEER values were measured for the vascular channel using the low-TEER setting, which is suitable for ECs, per the manufacturer.

### Immunofluorescence and Microscopy

#### Staining of Monolayer ECs, Pericytes, and Astrocytes

For TJ staining, ECs were fixed with ice-cold ethanol for 30 minutes and acetone for 1 minute. For all other staining, ECs, pericytes, and astrocytes were fixed in 4% PFA for 15 minutes. Fixed monolayers were then washed twice in PBS, blocked for an hour in blocking buffer (5% goat or donkey serum and 0.3% Triton X-100 in PBS), and incubated overnight with appropriate primary antibodies in a diluting solution at 4 °C (5% goat or donkey serum and 0.01% Triton X-100 in 1× PBS). The following day, cells were stained with secondary antibodies in 1× PBS for 1 hour at room temperature and DAPI (1:5000) for 10 minutes. Imaging was performed on a ZEISS LSM700 confocal microscope using ×10 (Plan Apochromat ×10 objective lens) and ×20 (Plan Apochromat ×20 objective lens).

#### Immunostaining of 3D MIMETAS Cultures

On day 10 post-seeding, MIMETAS chips were fixed with either 4% PFA for 15 minutes for standard staining or ethanol/acetone for TJ visualization. For fixation and blocking, the solution was introduced as follows: 100 µL in the right channel inlet, 50 µL in the right channel outlet, 50 µL in the left channel inlet, and 50 µL in the left channel outlet. The plate was placed on the OrganoFlow for rocking during staining. Chips were blocked for an hour in a blocking buffer and incubated overnight with primary antibodies in a diluting solution at 4 °C as follows: 25 µL in the right channel inlet and outlet, and 15 µL in the left channel inlets and outlets. Cells were washed and incubated with secondary antibodies and DAPI as above. Then, chips were postfixed with 4% PFA for 15 minutes before microscopic imaging.

### Bulk RNA Sequencing, Alignment, and Analysis

Total RNA was extracted as previously described.^24^ RNA samples underwent quality assessment using the Qubit 2.0 Fluorometer, and RNA integrity was assessed using the Agilent TapeStation 4200. Subsequently, RNA sequencing libraries were prepared using the NEBNext Ultra II RNA Library Prep Kit following the manufacturer’s protocol. The samples were sequenced in a 2x150bp Paired-End configuration using the Illumina NovaSeq instrument. Previously published samples were obtained from Gene Expression Omnibus data sets, including GSE138025, GSE40291, GSE57662, GSE131039, GSE97575, GSE73721, GSE97324, GSE138025, GSE122588, GSE129290, GSE97100, GSE126449, GSE108012, GSE151976, GSE85403, GSE82207, GSE77250, GSE82207, GSE157852, GSE243193, GSE225549, and GSE227501. Quality control for the samples was conducted using FastQC,^53^ followed by adapter trimming with Skewer (version 0.2.2).^54^ The resulting reads were aligned to the human reference genome (hg38) using the STAR aligner,^55^ and counts were generated using Subread.^56^ Normalization was performed using the trimmed mean of M-values method implemented in the edgeR package.^57^ Fragments per kilobase of transcript per million mapped reads and Log_2_ counts per million matrices were generated to account for library size variations. Differential expression analysis was performed using the edgeR package, considering *P*_adj_<0.05 and a log_2-_fold change <−0.25 or >0.25. Adjusted *P* values were calculated using the Benjamini-Hochberg procedure implemented in edgeR. Principal component analysis was performed, and Pearson correlation values were computed using the resulting Log_2_ counts per million values. Heatmaps were generated with the pheatmap package^58^ using min-max scaling on normalized counts.

### Principal Component Analysis From Single-Cell RNA-Seq Databases

Human single-cell RNA sequencing data sets were collected from multiple publicly available resources.^59–78^ All data sets were downloaded and subjected to quality control using the Seurat, version 5.1.0, R package.^79^ ECs were identified, captured, and subsequently isolated across all data sets. To generate pseudobulk profiles, the AggregateExpression function was utilized, and raw count data were exported. Following pseudobulk data generation, principal component analysis was performed, and Pearson correlation coefficients were computed based on Log_2_-transformed counts per million values.

### Gene Set Enrichment Analysis

Trimmed mean of M-values–normalized counts obtained from edgeR were directly incorporated into the standard Gene Set Enrichment Analysis (GSEA)^80^ workflow. Novel gene sets associated with pathways, biological processes, and specific cell types of interest, including VEGF-A signaling, canonical Wnt/β-catenin signaling, noncanonical Wnt signaling, chemokine-cytokine signaling, TGF-β signaling, ECM, cell-cell adhesion, transporters, Notch signaling, angiogenesis, EC proliferation, EC migration, apoptosis, antigen processing and presentation, BBB, inflammation, EndoMT (endothelial-to-mesenchymal transition), and tip cells, were compiled as part of the GSEA analysis (Data Set S1H). These gene sets were sourced from the Gene Ontology,^81^ KEGG (Kyoto Encyclopedia of Genes and Genomes),^82^ DAVID (Database for Annotation, Visualization, and Integrated Discovery),^83^ and GSEA^80^ databases, as previously described.^84^ The ranked list of differentially expressed genes, along with the curated gene sets, was uploaded into GSEA 4.3.2 software and analyzed using the Gsea module with the following settings: number of permutations=10 000; collapse/remap to gene symbols=no_collapse; permutation type=gene set; enrichment statistic=classic; and maximum gene set size=1500. All other parameters were kept at their default values. Gene sets with a false discovery rate q value ≤0.01 were classified as significantly enriched (na_pos) or depleted (na_neg).

### Statistical Analysis

Data were analyzed using GraphPad Prism. Individual wells, inserts, or chips were considered biological replicates. All key experiments were repeated independently, and detailed information about sample size, error bars, and the number of experiments is provided in each figure legend, while *P* values are provided in the figure. For comparisons between 2 groups in which data were normally distributed, we performed an unpaired Student *t* test if homogeneity of variance was satisfied or an unpaired *t* test with the Welch correction if homogeneity of variance was not satisfied. For comparisons between >2 groups in which data were normally distributed, we performed an ordinary 1-way ANOVA if homogeneity of variance was satisfied or a Welch ANOVA if homogeneity was not satisfied. For comparisons between >2 groups in which homogeneity of variances was satisfied but data were not normally distributed, we performed the Kruskal-Wallis test. For experiments with 2 independent variables, a 2-way ANOVA with the Bonferroni post hoc test was performed. A repeated measures 2-way ANOVA was utilized to assess changes to wound area at multiple timepoints in the wound scratch assay in Figure 1I. *P*<0.05 was considered statistically significant.

## Results

### Reprogramming iPSC-Derived bpECs Into rBMECs

During early mammalian embryonic development, the mesodermal layer gives rise to generic ECs. These generic ECs surround the neural tube and generate the CNS vasculature under the guidance of VEGF-A (vascular endothelial growth factor A) and Wnt/β-catenin signaling. We hypothesized that iPSC-derived rBMECs could be generated using a 2-step approach mirroring CNS vascular development (Figure 1A). In the first step, iPSCs were differentiated into bpECs by sequential exposure initially to BMP4 (bone morphogenic protein 4)/bFGF (basic fibroblast growth factor) to generate a mesodermal intermediate followed by VEGF-A/thymosin-β4 using a published protocol with minor modifications^35^ (Figure 1A). bpECs were purified from other mesodermal cells by high expression for CD31^+^ (platelet/EC adhesion molecule [PECAM1/CD31])/VE-cadherin^+^ (vascular endothelial cadherin, CDH5) using FACS on day 10 of the differentiation (sort A) and expanded on collagen IV/fibronectin-coated dishes in human endothelial serum-free media (Figure 1A; Figure S1A; see Methods section). Sort A efficiency was similar across all tested iPSC lines (IMR90, FA0000010 [FA10], and NCRM-5; Figure S1C) in line with previously published iPSC-derived EC protocols.^85–87^ In the second stage, bpECs were transduced with lentiviruses expressing FOXF2 and ZIC3. Transduced and reprogrammed bpECs were expanded and repurified by FACS (fluorescence activated cell sorting; sort B) to generate a pure population of rBMECs that could be expanded reliably for 5 passages after purification (Figure 1A; Figure S1A). After sort B, rBMECs expressed higher levels of CD31 and VE-cadherin proteins on the cell surface compared with bpECs (Figure 1B and 1E) with comparable efficiency across iPSC lines (Figure S1D).

### rBMECs Express Key EC and BBB Markers and Respond to Angiogenic Stimuli

During barrier genesis, brain ECs express a unique repertoire of TJ proteins and transporters, and decrease caveolar-mediated transcellular transport to protect the brain parenchyma from exposure to various substrates and toxins. To assess rBMECs for structural and functional BBB properties, we first examined the expression of several EC and BBB proteins. The bpECs and rBMECs derived from all 3 iPSC lines expressed high levels of key EC identity proteins (eg, VE-cadherin and CD31; Figure 1B and 1E) and BBB TJ proteins (CLDN-5, OCLN, and ZO-1 [zonula occludens-1]) by immunofluorescence staining (Figure 1C and 1D; Figure S1E through S1J). rBMECs had higher CLDN-5 and OCLN protein levels compared with bpECs by Western blot (Figure 1F and 1G). In addition, rBMECs expressed lower total CAVEOLIN-1 protein compared with bpECs although these levels were higher than those present in primary HBMECs (Figure S1K and S1M). The ratio of the phosphorylated to total CAVEOLIN-1 proteins, a proxy for transcellular transport, was lower in rBMECs compared with bpECs and equivalent to that of HBMECs (Figure S1K and S1N). rBMECs also expressed similar levels of ABCC4 (ATP-binding cassette sub-family C member 4) transporter protein compared with bpECs (Figure S1L and S1O). Importantly, rBMECs did not express EpCAM (epithelial cell adhesion molecule) on their surface by flow cytometry (Figure S1B), indicative of an endothelial identity.

Angiogenesis occurs before barriergenesis and decreases during CNS development. To assess the angiogenic ability of rBMECs, we measured the proliferative index of hiPSC-derived ECs by immunofluorescence staining for Ki67 and DAPI. rBMECs proliferated at a similar rate to bpECs but at a lower rate than primary HBMECs when cultured in the absence of growth factors (VEGF-A and bFGF; Figure 1H). To assess the response to angiogenic stimuli, we performed a wound scratch assay on EC monolayers (bpECs, rBMECs, and HBMECs) treated with either basal media or media supplemented with an angiogenic cocktail. We analyzed the area of wound closure over time as a readout of cell migration and proliferation and calculated the angiogenic potential (see Methods section; Figure 1I and 1J; Figure S1P through S1Q). By 36 hours, rBMEC migration increased in response to the angiogenic cocktail compared with control conditions (Figure 1I). Primary HBMECs and rBMECs exhibited a comparable angiogenic potential, while bpECs responded more strongly to the angiogenic stimuli (Figure 1J). In summary, rBMECs express both key EC and BBB proteins, and proliferate and migrate in response to angiogenic stimuli similar to primary HBMECs, which is a fundamental feature of EC identity.

### Transcriptome Profiling Reveals That rBMECs Express a Subset of the BBB Gene Repertoire

To better characterize the brain-specific EC identity and BBB gene expression repertoire of rBMECs, we performed bulk RNA sequencing (bulk RNA-seq) studies. We used principal component analysis to assess the brain specificity of the rBMEC transcriptome by comparing our data set to the published transcriptome data sets of human peripheral ECs generated in vitro or isolated from specific organs in vivo including cardiac, dermal, pulmonary, and aortic ECs.^24,88–90^ Based on this analysis, rBMECs clustered closely with bpECs, while primary HBMECs clustered more closely with ECs derived from other organs/tissues (Figure 2A; Data Set S1A). Next, we compared published transcriptome data sets of brain ECs from fetal, adult, and aged samples^59–63,66–73,75–78,91,92^ with that of rBMECs to approximate their level of developmental maturity. rBMECs, along with bpECs, cluster more closely to fetal brain ECs and brain organoid ECs and further away from aged brain ECs, suggesting that they are immature in development, similar to other iPSC-derived cells (Figure 2B; Data Set S1B). Finally, we compared the rBMEC transcriptome with that of previously published iBMECs,^24^ which verified that rBMECs are not epithelial in nature (Figure S2A; Data Set S1C). These transcriptomic analyses confirm that rBMECs have a brain endothelial identity and resemble fetal brain ECs.

We performed differential gene expression analysis between rBMECs and HBMECs, or bpECs, to identify which brain EC identity and BBB gene signatures are distinct among them. Many BBB-relevant genes were differentially expressed between rBMECs and HBMECs (Figure 2C; Data Set S1D), and rBMECs and bpECs (Figure S2B; Data Set S1F). Gene Ontology analysis revealed that pathways related to blood vessel development, vasculature development, maintenance of BBB permeability, and regulation of blood vessel EC migration were upregulated in rBMECs compared with HBMECs (Figure 2D; Data Set S1E). Several BBB genes encoding for TJs (eg, CLDN-5, OCLN, and JAM2 [junctional adhesion molecule 2]), transporters (eg, SLC2A1 [solute carrier family 2 member 1], ABCC4, and SLC1A1 [solute carrier family 1 member 1]), receptor-mediated transcytosis (eg, CAV1, CAV2 [caveolin-2], and LRP10 [LDL receptor related protein 10]), and ECM components (eg, COL4A1 [collagen type IV alpha 1 chain] and COL4A2 [collagen type IV alpha 2 chain]) were higher in rBMECs compared with HBMECs (Figure 2E through 2G; red); however, other BBB genes within these categories were either not different or lower in expression (Figure 2E through 2G, black and blue). In addition, rBMECs expressed higher levels of brain EC identity markers (eg, FOXF2, JAG2 [jagged canonical notch ligand 2], SPOCK2 [SPARC, cwcv and kazal like domains proteoglycan 2], TNFRSF19 [NF receptor superfamily member 19], PROM1 [prominin 1], and APCDD1 [adenomatosis polyposis coli down-regulated 1]) compared with primary HBMECs (Figure 2H), suggesting a closer proximity to a brain EC identity. Although rBMECs shared a similar gene expression profile with bpECs (Figure S2B), several key BBB genes (eg, CDH5, APCDD1, SPOCK2, CLDN-5, OCLN, LRP8 [low-density lipoprotein receptor related protein 8], and JAG2 [jagged canonical notch ligand 2]) were upregulated significantly in rBMECs compared with bpECs (Figure S2C through S2F), suggesting improvement in some BBB gene signatures. Moreover, rBMECs showed reduced expression of LAMs (eg, VCAM-1 [vascular cell adhesion molecule -1] and SELP [P-selectin]) compared with either HBMECs or bpECs, indicative of a more mature brain EC identity. We then analyzed the expression of specific zonation markers across arterial, capillary, and venous ECs (see Methods section). Compared with HBMECs, the rBMEC transcriptome showed higher expression of capillary (139) and arterial (115) markers and fewer venous (71) markers (Figure S2G), suggesting that rBMECs have a mixed arterial and capillary zonation identity at the population level. In summary, the rBMEC transcriptome shows stronger expression of several brain EC identity and BBB gene signatures compared with primary HBMECs and bpECs.

### rBMECs Have Robust BBB Functional Properties

During BBB maturation, brain ECs reduce their paracellular and transcellular permeability via expression of TJ proteins, reduced caveolar-mediated transcytosis, and upregulation of efflux transporters. Therefore, we compared these BBB functions in rBMECs, bpECs, and primary HBMECs. First, we measured TEER over time as a readout of the paracellular EC barrier formed by TJs. IMR90-derived rBMECs had a higher average TEER (40.5±4.2 Ω*cm^2^) compared with either bpECs (25.0±0.4 Ω*cm^2^) or primary HBMECs (24.7±3.0 Ω*cm^2^). The area under the curve for the last 100 hours of TEER recording was 2-fold higher in rBMECs compared with HBMECs (Figure 3A and 3B). TEER was also increased in rBMECs derived from 2 other hiPSC lines (FA10 and NCRM-5) compared with primary HBMECs (Figure S3A, S3B, S3E, and S3F). Next, we used 3 different molecular weight tracers, sodium fluorescein (=376 Da), and 3- and 70-kDa dextrans to assess their diffusion across the EC monolayer in a transwell system. rBMECs showed a reduced Pe for the smallest tracer (sodium fluorescein) across the monolayer compared with bpECs and HBMECs, indicative of a tighter paracellular barrier (Figure 3C). Although HBMECs showed a significantly higher Pe compared with rBMECs for the medium-sized tracer (3-kDa dextran), there was no difference between rBMECs and bpECs (Figure 3D). In contrast, the Pe for the 70-kDa dextran was low across all 3 EC types (rBMECs, bpECs, and HBMECs), suggesting low transcellular-mediated transport (Figure S3C and S3D). Overall, these functional data demonstrate that rBMECs have a tighter paracellular barrier compared with bpECs and primary HBMECs.

To assess transcellular rBMEC barrier properties, we examined caveolar-mediated transcytosis by incubating EC monolayers with a fluorescently labeled BSA (molecular weight, MW=60 kDa) and measuring its uptake inside cells by flow cytometry. rBMECs and bpECs showed very small changes in the MFI of intracellular fluorescently labeled BSA compared with HBMECs (Figure 3E and 3F), indicative of low caveolar-mediated uptake. Finally, we assessed the function of PGP (P-glycoprotein encoded by the ABCB1 [ATP-binding cassette subfamily B member 1]), a transporter that effluxes drugs from the brain to the blood.^93^ We incubated HBMEC, bpEC, and rBMEC monolayers with Rhodamine 123, a compound that is pumped out of the brain ECs by PGP,^94^ in the presence or absence of a PGP-specific inhibitor, PSC-833. Cells were subsequently analyzed by flow cytometry to assess changes in the MFI of Rhodamine 123, and we calculated the efflux rate based on the MFI change in the presence/absence of the inhibitor as a readout of efflux transporter activity. HBMECs, bpECs, and rBMECs had comparable efflux rates in response to PSC-833, suggesting similar PGP activity (Figure 3G and 3H). However, rBMECs had a lower fold change in Rhodamine 123 MFI compared with HBMECs (Figure 3I), suggesting an overall greater efflux of the dye. These data suggest that rBMECs have an increased efflux of Rhodamine 123 compared with bpECs and HBMECs although PGP activity is similar in all 3 EC subtypes.

### Modeling the Human 3D NVU/BBB Using rBMECs and hiPSC-Derived Astrocytes and Pericytes

The acquisition and maintenance of the BBB occur through local interactions of brain ECs with mural cells and astrocytes via the ECM. We investigated whether rBMECs can interact with iPSC-derived astrocytes and pericytes to generate a fully iPSC-derived human NVU in a 3D microfluidic system (Figure 4A). We individually differentiated iPCs from a neural crest intermediate following an established protocol with minor modifications^44^ (Figure S4A and S4C) and reprogrammed iACs from NPCs (neural progenitor cells) as previously described^46^ (Figure S4B and S4D). Then, we cocultured all 3 NVU cell types using the microfluidic 3-lane MIMETAS 64-chip OrganoPlate.^95,96^ rBMECs or HBMECs were introduced on day 0 in the right blood channel against a collagen I/IV gel in the middle ECM channel to enable formation of EC tubules. iACs and iPCs were introduced in the left brain channel after 2 days, and the system was allowed to mature for 10 days with bidirectional flow in a rocking platform^95,96^ (Figure 4A). By 10 days, rBMECs formed continuous tubules, and iACs and iPCs migrated toward the blood vessel channel to make contact with the brain EC tubules (Figure 4B). rBMEC tubules were positive for the CD31, VE-cadherin, and BBB TJ proteins, CLDN-5 and ZO-1 (Figure 4C; Videos S1 and S2). In 3D cocultures of rBMECs with iACs and iPCs, ZO-1^+^ ECs were in close proximity to S100β^+^ (astrocyte) and PDGFRβ^+^ (pericyte) cells, suggesting that iACs and iPCs migrated across the middle ECM channel to contact the rBMEC tubules, which had formed an open lumen (Figure 4D and 4E; Videos S3 and S4). Therefore, rBMECs can form tubules and interact with iPSC-derived astrocytes and pericytes to generate a 3D human NVU that incorporates flow in the MIMETAS platform.

To determine whether rBMEC tubules within the 3D microfluidic system showed improved BBB properties when cultured with iPCs and iACs, we performed tracer diffusion studies using small (biocytin, 890 Da) and large (70-kDa dextran) tracers. We introduced the tracers into the EC tubules in the blood channel, allowed them to diffuse across the EC tubules, and measured the normalized fluorescence intensity in the middle ECM channel as a readout of tracer leakage through the EC tubule at 3 distinct time points (15, 30, and 45 minutes). rBMEC tubules exhibited a significantly lower leakage for both biocytin and 70-kDa dextran compared with primary HBMEC tubules, indicative of tighter barrier properties (Figure 4F through 4H). Moreover, rBMEC tubules cocultured with iACs and iPCs exhibited lower permeability to biocytin compared with rBMEC tubules cultured alone for 45 minutes, suggesting that iPCs/iACs increase the barrier properties of rBMEC tubules (Figure 4H). We then measured TEER across the EC tubule in the 3D microfluidics system using the OrganoTEER instrument from MIMETAS.^95^ While there was some variability across experiments consistent with prior published studies in this 3D system,^97,98^ rBMEC tubules displayed, on average, a higher TEER compared with HBMEC tubules alone (Figure 4I). rBMEC tubules also had a significantly higher TEER when cocultured with iACs/iPCs (Figure 4I), consistent with reduced tracer diffusion across the 3D iPSC-derived NVU system (Figure 4H). Thus, rBMECs form stable and continuous tubules in the 3D microfluidic MIMETAS system, and they interact with iPCs/iACs to form an iPSC-derived NVU network with tighter BBB properties resembling more closely the human BBB properties in vivo.

Finally, we compared transcriptome changes in rBMECs after culture in the 3D microfluidic system with those in a monolayer culture (2-dimensional). We collected mRNA from 3D rBMEC tubules cultured alone in the MIMETAS for bulk RNA sequencing and compared their transcriptomes to rBMEC samples cultured in static 2D monolayers from Figure 2 (Figure S4E). A subset of BBB-related genes was upregulated in rBMECs after 3D culture including TJ proteins primarily of the MARVEL domain-containing protein (TAMP) subfamily, which are highly expressed by brain ECs (TJP1 [tight junction protein 1], OCLN, MARVELD2 [MARVEL domain containing 2; tricellulin], and CGNL1 [cingulin-like 1]), the regulator of caveolar-mediated transcellular transport MFSD2A (major facilitator superfamily domain-containing protein 2A),^99^ several transporters for the thyroid hormone, large neutral amino acids and anions (eg, SLC16A2 [solute carrier family 16 member 2) and SLC7A5 (solute carrier family 7 member 5), and brain EC identity genes (eg, FOXF2, TNF4SF19 [tumor necrosis factor receptor superfamily member 19], TBX1 [T box transcription factor 1], and MARVELD2; Figure S4F and S4H through S4K). Furthermore, pathways related to EC proliferation, angiogenesis, TJs, adherens junctions, and noncanonical Wnt signaling were also upregulated in 3D-cultured rBMEC tubules compared with 2D rBMEC monolayers by GSEA using a BBB-specific gene list^84^ (Figure S4G). This significant transcriptome shift suggests that rBMECs respond robustly to shear stress to upregulate a subset of brain EC identity and BBB-related genes. The upregulation of pathways relating to angiogenesis and proliferation aligns with a prior study showing that shear stress increases expression of angiopoietin-2.^100^ Finally, the upregulation of tight and adherens junctions, and other BBB-related genes suggests that rBMECs strengthen their barrier properties in the 3D system.

### Utilizing rBMECs to Examine Brain EC Dysfunction in Alzheimer disease

In neurodegenerative diseases such as Alzheimer disease, BBB dysfunction exacerbates disease pathogenesis. However, brain ECs may also be vulnerable at earlier stages of disease, before overt neurodegeneration and BBB leakage.^101^ We investigated how an fAD mutation may affect BBB function by generating rBMECs from hiPSCs in which an autosomal dominant mutation *APP*^*Lon*^ (London; *APP V717I*) was introduced via CRISPR (clustered regularly interspaced short palindromic repeats) technology.^38,102^ Causative of early onset fAD, *APP^Lon^*, is associated with cerebral amyloid angiopathy in patients and transgenic mouse models.^103,104^ We compared the TEER of rBMECs carrying 1 or 2 copies of *APP V717I* to that of isogenic control rBMECs, which revealed that *APP^*Lon*^* rBMECs had reduced TEER (Figure 5A and 5B), indicative of reduced barrier integrity. Simultaneously, we performed bulk RNA sequencing to compare the transcriptomes of *APP^*Lon*^* and isogenic control rBMECs. *APP^*Lon*^* rBMECs showed upregulated expression of many inflammatory pathways (Figure 5C) and several inflammatory cytokines and a subset of LAMs (Figure S5A). These data demonstrate that the *APP*^*Lon*^ mutation drives both upregulation of neuroinflammatory signatures and barrier dysfunction in rBMECs.

Because most patients with Alzheimer disease have amyloid deposits in the brain vasculature,^105–107^ we investigated whether treatment of control rBMECs with low concentrations of pathogenic oAβ42 could reproduce the effects seen with *APP*^*Lon*^ because this mutation affects Aβ processing. First, we assessed whether oAβ42 affects the paracellular barrier properties of rBMECs. In contrast to published studies showing that treatment with high doses of oAβ42 (10 µM) decreases the TEER of brain ECs,^108^ we observed no change in TEER over 72 hours when rBMECs were exposed to either 500-nmol/L or 1-µM oAβ42 (Figure 5D and 5E). However, 2 proinflammatory cytokines, TNFα (tumour necrosis factor-alpha) and IL1β (interleukin-1 beta), significantly reduced rBMEC TEER over 24 hours, confirming that control rBMECs can respond to inflammatory molecules to increase their barrier permeability (Figure S5C and S5D). Therefore, rBMECs can model both barrier-disruptive conditions driven by neuroinflammation (TNFα and IL1β) or genetic drivers (*APP*^*Lon*^).^102^ Moreover, these findings suggest that *APP^*Lon*^* affects brain EC barrier properties independently of Aβ accumulation, similarly to what has been described in APP mutant hiPSC–derived neuronal models.^109–111^

To determine if oAβ42 affects EC inflammatory activation, we measured changes in LAMs and chemokines via qPCR. VCAM-1 and ICAM-1 (intercellular adhesion molecule-1) were increased after 72 hours of treatment with 1-µM oAβ42 (Figure S3G). CCL2 (C-C motif chemokine ligand 2), a key leukocyte attractant,^112^ was also upregulated in rBMECs (Figure S5B). Therefore, rBMECs respond to oAβ42 by upregulating expression of LAMs and proinflammatory cytokines/chemokines. To examine the acute effects of oAβ42, we treated rBMECs with 500-nmol/L oAβ42 for 6 hours and analyzed changes in LAMs and proinflammatory cytokines/chemokines, as possible downstream effectors of oAβ42-mediated BBB dysfunction. oAβ42 increased mRNA expression of ICAM-1, VCAM-1, E-SELECTIN, *CCL2*, IL-1*β*, IL-6 (interleukin 6), and TNF*α* within 6 hours (Figure 5F). In addition, ICAM-1 and VCAM-1 total and surface protein levels assessed by Western blotting and flow cytometry, respectively, were upregulated in rBMECs after acute oAβ42 treatment (Figure 5G through 5I). Finally, we examined potential signaling pathways that might contribute to oAβ42-induced inflammatory phenotypes in rBMECs. Phosphorylation of STAT3 (signal transducer and activator of transcription 3), a key mediator of proinflammatory processes in brain ECs,^113,114^ was elevated in rBMECs treated with oAβ42 (Figure S5E and S5F). Therefore, β-amyloid peptides may act as a proinflammatory stimulus in rBMECs to upregulate expression of a subset of the EC inflammatory gene repertoire relevant for increased interactions with immune cells. Overall, these data show that rBMECs have strong functional BBB properties and are suitable to study neuroinflammatory and neurodegenerative states, suggesting that they could be a valuable model for future investigations of BBB contribution to disease.

## Discussion

The generation of an in vitro human NVU system with strong BBB properties to model neurological diseases and develop effective therapeutics for CNS delivery has been a major technological hurdle for many decades.^10–13^ While several HiPSC-derived BBB models have been developed in the past decade, their utility has been limited by a lack of brain-specific EC identity and the inability to replicate the multifaceted properties of the human BBB. Prior methods have either generated ECs with limited BBB properties,^26,35^ inconsistent immune responses,^18,19,22,23,26^ generic but not brain-specific EC identity,^24,32^ or mixed epithelial-EC identity that show robust barrier properties due, in part, to their epithelial cell nature.^17–20,22,23,50^ In this study, we have generated ECs with improved brain-specific EC identity and strong BBB properties through a strategy that combines differentiation of hiPSCs into bpECs followed by overexpression of 2 brain-specific transcription factors, FOXF2 and ZIC3, to drive brain-specific EC identity. These rBMECs express higher levels of key brain EC identity markers, and several BBB markers, including TJ-associated proteins and transporters at both transcript and protein levels, can respond to angiogenic stimuli and respond robustly to oAβ42 treatment by upregulating an inflammatory gene signature. At a functional level, rBMECs have a tighter paracellular barrier, reduced caveolae-mediated transport, and equivalent PGP activity compared with primary HBMECs. Finally, rBMECs can interact with iPSC-derived pericytes and astrocytes in a 3D system with flow and strengthen their barrier properties through cell-cell interactions, mirroring the 3D NVU organization in the human brain. Thus, rBMECs generated through this new approach can be utilized as an in vitro human BBB monolayer model and a 3D microfluidic NVU model in combination with iPSC-derived pericytes/astrocytes in the MIMETAS platform. This highly tractable brain EC model may be applied for studying mechanisms of disease and therapeutic development.

Why are both FOX2 and ZIC3 overexpression critical for the acquisition of the brain EC identity? A prior study by Roudnicky et al^32^ overexpressed either FOXF2 or ZIC3 through adenoviral transduction in generic ECs generated by a different method. They found that FOXF2 overexpression improved some BBB properties but also upregulated inflammatory gene signatures.^33^ In contrast, ZIC3 overexpression had no effect on the barrier properties of ECs.^33^ Contrary to these studies, we find that simultaneous overexpression of FOXF2 and ZIC3 does not induce inflammatory gene signatures. For example, LAMs such as SELP and VCAM-1 were downregulated, whereas SELE and ICAM-1 were unaltered in rBMECs compared with primary HBMECs. Moreover, FOXF2/ZIC3 combination drives the acquisition of a larger BBB transcriptome repertoire, albeit incomplete, in rBMECs with improved BBB functional properties such as a stronger paracellular barrier regulated by TJs and suppression of LAMs critical for immune cell trafficking into the CNS. However, the precise mechanism by which the FOXF2/ZIC3 combination reprograms bpECs to strengthen their brain EC identity and BBB properties remains unclear. We postulate that bpECs generated via the Praca et al^35^ protocol are more amenable to acquiring stronger BBB properties than other EC subtypes. Therefore, FOXF2 and ZIC3 reprogramming seems more effective in ECs that exhibit some BBB-like properties but remain plastic in the acquisition of their final fate.

FOXF2 and ZIC3 function downstream of Wnt/β-catenin signaling,^31^ a pathway critical for BBB development.^34^ However, treatment of rBMECs with CHIR99021, a potent activator of Wnt/β-catenin signaling, did not have an additive effect on the acquisition of BBB barrier properties (data not shown). This was somewhat surprising because a recent study showed that a small molecule cocktail (cARLA) that activates Wnt/β-catenin and cAMP (cyclic adenosine monophosphate) signaling and inhibits TGF-β signaling in generic ECs^27^ was sufficient to induce robust BBB properties (tight barrier and high efflux activity).^27^ However, improvements in BBB function were not accompanied by increased expression of FOXF2, ZIC3, and several other BBB genes (eg, ITM2A [integral membrane protein 2A] and OCLN) that are robustly upregulated in rBMECs. Nevertheless, cARLA favorably increased expression of the efflux transporter ABCB1 (ATP-binding cassette subfamily B member 1), which was lower in the rBMEC 2D monolayer. These 2 diverse strategies (cARLA versus FOXF2/ZIC3 reprogramming) may induce complementary BBB transcriptome repertoires and functional properties, with FOXF2 and ZIC3 reprogramming upregulating essential brain EC identity markers. However, neither strategy was able to induce high expression of a key BBB transporter MFSD2A, essential for suppression of transcytosis.^99^ Future studies will determine whether a combination of small molecules with FOXF2/ZIC3 reprogramming strategies can enhance the brain EC identity of BMECs with a more complete set of the mature BBB transcriptome.

Several static^115,116^ and perfusable^17,20,117–120^ iPSC–derived 3D NVU models have been generated, with conflicting reports on whether shear stress can induce transcriptomic changes in iBMECs. In one microfluidic system, exposure to 4 or 12 dynes/cm^2^ of shear stress had a limited effect on TJ expression in iBMECs.^119^ In another study, treatment of iBMECs with 0.5 or 2.4 dynes/cm^2^ upregulated TJP1 and OCLN expression but also downregulated other key TJs such as CLDN-5, increased inflammatory adhesion proteins such as VCAM-1, and increased expression of CAV1 and CAV2.^20^ In contrast, our transcriptome analysis show that rBMEC tubules respond to shear stress by upregulating several brain EC identity genes in the 3D microfluidic system with a bidirectional shear stress estimated at 0 to 1.4 dynes/cm^2^.^121^ In our system, we observed similar increases in TJP1 and OCLN without changes in CLDN-5, CAV1, CAV2, or VCAM-1. Importantly, rBMECs upregulated expression of key BBB genes such as FOXF2 and MFSD2A although overall MFSD2A levels remain relatively low. These data suggest that 3D culture and shear stress can drive a subset of BBB properties resembling more closely the in vivo counterpart. We anticipate that the addition of iPSC-derived pericytes and astrocytes may push the BBB transcriptome of the 3D NVU system closer to the in vivo system, which will be the focus of future studies.

Here, we also investigated the effect of fAD mutation *APP*^*Lon*^ on barrier properties of rBMECs and compared this mutation to the response of rBMECs to oAβ42 peptides. In contrast to published studies,^108^ oAβ42 did not affect barrier tightness of rBMECs but was sufficient to upregulate significantly the expression of proinflammatory cytokines/chemokines and LAMs, including VCAM-1 and ICAM-1. These data are consistent with recent findings that primary HBMECs upregulate VCAM-1, ICAM-1, E-SELECTIN, and CCL2 after treatment with conditioned media from hiPSC-derived cortical neurons harboring the *APP*^*Swe/Swe*^ mutation (*KM670/671NL*).^122^ We used a lower, more physiologically relevant dosage of oAβ42 than described,^108^ which is nontoxic to rBMECs and may explain the lack of effect on the barrier properties. In contrast, the fAD mutation *APP*^*Lon*^ disrupted the paracellular barrier of rBMECs, suggesting that *APP*^*Lon*^ acts independently of β-amyloid accumulation to drive BBB dysfunction. Disentangling these mechanisms further will be the focus of future studies.

While our transcriptomic analysis suggests that rBMECs are relatively developmentally immature, they can still be used to study intrinsic genetic risk factors or extrinsic environmental factors critical for postnatal brain development and neurodegenerative processes, such as Alzheimer disease. rBMECs interact very closely with iPSC-derived pericytes and astrocytes. Because astrocytes mature and ensheath brain ECs postnatally^123^ and this process correlates with increased expression of several BBB transporters,^124^ the 3D model system can be used to study postnatal brain development with the addition of other relevant cell types (eg, neurons). In addition, rBMECs can be used to test how genetic factors (eg, *APP*^*Lon*^ mutation) or extrinsic environmental factors present in neurodegeneration (eg, oAβ42) affect brain EC function. While the maturity of rBMECs may limit their ability to model aged ECs, recent studies demonstrate that brain ECs can reactivate developmental pathways, immune responses, and angiogenesis pathways in either vascular pathologies^62^ or neuroinflammatory CNS diseases^84^ resembling their fetal counterparts; hence, immature brain ECs can provide useful insights for disease modeling. Overall, we have shown that rBMECs can be used successfully to model neuroinflammatory diseases and barrier-disrupting states. Taken together, we have generated and characterized at the molecular and functional levels an EC subtype similar in identity to brain ECs that can be used either alone or with other NVU cells to study pathogenic mechanisms of neurological diseases and to develop efficient methods for drug delivery to the CNS.

### Limitations of This Study

While rBMECs have robust brain EC molecular identity and BBB functional properties, they do not fully recapitulate all aspects of the BBB transcriptome including expression of some BBB-specific transporters such as MSFD2A. Furthermore, lentiviral-mediated overexpression of FOXF2 and ZIC3 may present challenges in reproducibility due to variable lentiviral efficiency and triggering potential antiviral immune responses. It should also be noted that although multiple lots of primary HBMECs from a commercial source were used for comparison with bpECs and rBMECs, other groups have reported better properties of these cells than those reported here, which likely reflects variability among individual commercial lots. Despite these limitations, primary HBMECs are a more appropriate control to compare the rBMECs rather than iPSC-derived iBMECs generated by prior protocols^17–23^ because iBMECs have molecular and transcriptome features of epithelial cells.^24^

## Article Information

### Acknowledgments

The authors thank Raphael Lis for providing FOXF2 (forkhead box F2) and ZIC3 (zic family zinc finger 3) expression plasmids and Paroma Mallick in Alejandro Chavez’s laboratory for subcloning FOXF2 and ZIC3 into pLEX_307 plasmid. Flow cytometry and cell sorting experiments were performed in the Columbia Stem Cell Initiative Flow Cytometry Core Facility under the guidance of Michael Kissner.

### Author Contributions

A. Cui, R. Patel, P. Bosco, D. Agalliu, and A.A. Sproul designed the research study. A. Cui, R. Patel, P. Bosco, E. Richters, and P. Barrilero Delgado performed the experiments. A. Cui, R. Patel, P. Bosco, and U. Akcan analyzed the data. A. Cui, R. Patel, P. Bosco, D. Agalliu, and A.A. Sproul wrote and edited the manuscript. D. Agalliu and A.A. Sproul provided funding support for the study.

### Sources of Funding

This work was supported by grants from the National Heart, Lung, and Blood Institute (R61/R33 HL159949) and the National Institute on Aging (RF1AG078352). A. Cui, U. Akcan, and D. Agalliu are also supported by grants from the National Eye Institute (R01EY033994), the National Institute of Neurological Disorders and Stroke (R21NS130265), the International OCD Foundation, the PANDAS (Pediatric Autoimmune Neuropsychiatric Disorder Associated with Streptococcus) Network, the PANDAS Physician Network, and the Global Lyme Alliance. A.A. Sproul is also supported by the Henry and Marilyn Taub Foundation and the Thompson Family Foundation.

### Disclosures

None.

### Supplemental Material

Table S1

Figures S1–S5

Videos S1–S4

Data Set S1

Major Resources Table

## Supplementary Material
