# Supplementary material for: Generation of hiPSC-Derived Brain Microvascular Endothelial Cells Using Directed Differentiation and Transcriptional Reprogramming
Source: Arterioscler Thromb Vasc Biol. 2025 Nov 25;46(1):210–31. doi: 10.1161/ATVBAHA.125.323397 (PMC12721698; doi:10.1161/ATVBAHA.125.323397)
Supplement: Supplementary file 1 [file atv-46-210-s001.pdf]

# SUPPLEMENTAL MATERIALS

## Generation of hiPSC-derived brain microvascular endothelial cells using directed differentiation and transcriptional reprogramming

Aomeng Cui, Ronak Patel, Patrick Bosco, Ugur Akcan, Emily Richters, Paula Barrilero Delgado, Dritan Agalliu and Andrew A. Sproul

Corresponding author: Andrew A. Sproul, Ph.D.

**Email:** aas2003@cumc.columbia.edu

Figures S1 to S5
Table S1
Legends for Videos S1 to S4
Legend for Dataset S1
Major Resources Table

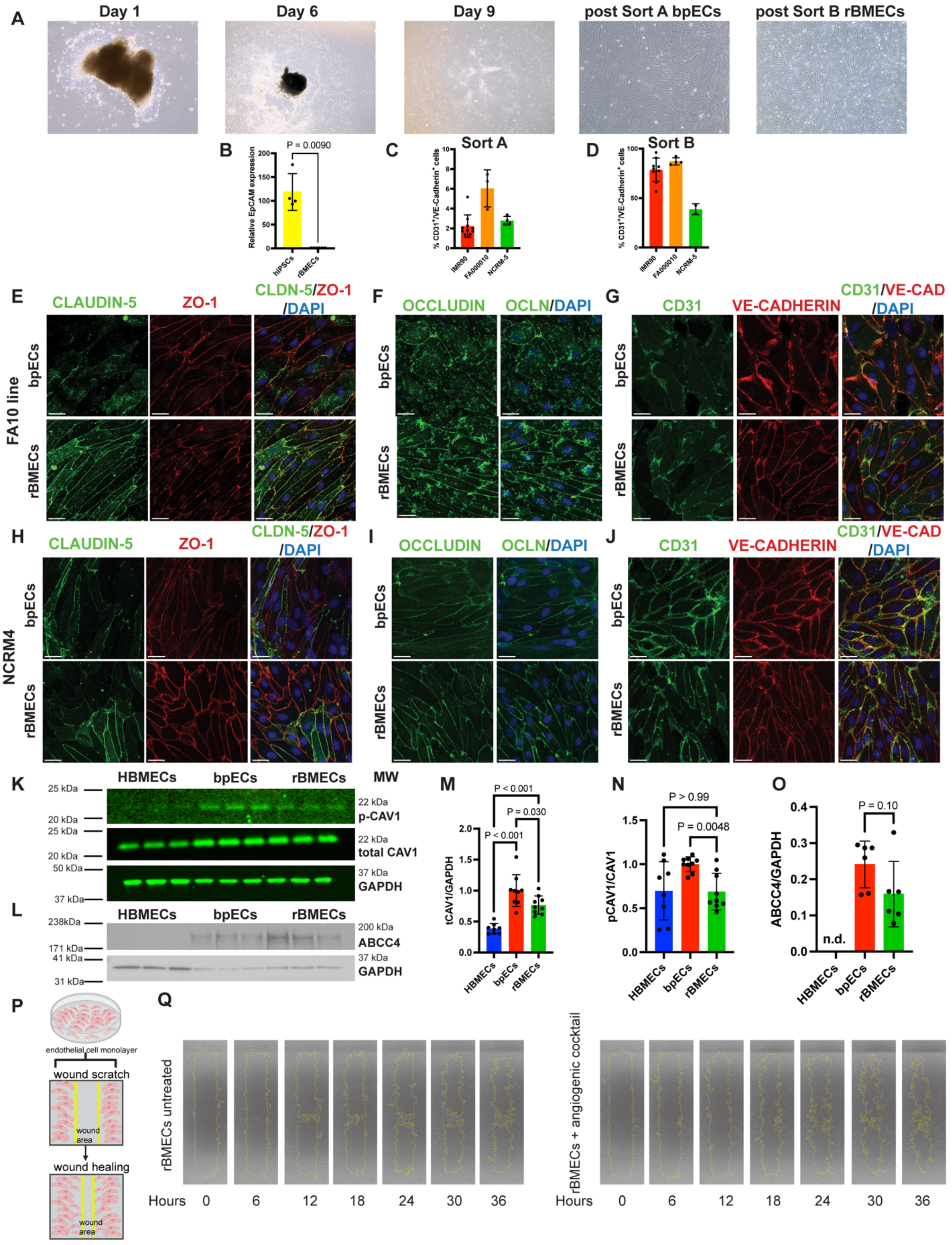

A
Day 1
Day 6
Day 9
post Sort A bpECs
post Sort B rBMECs
B
P = 0.0090
Relative EpCAM expression
hiPSCs
rBMECs
C
Sort A
% CD31+/VE-Cadherin+ cells
IMR90
FA000010
NCRM-5
D
Sort B
E
CLAUDIN-5
ZO-1
CLDN-5/ZO-1/DAPI
FA10 line
bpECs
rBMECs
F
OCCLUDIN
OCLN/DAPI
G
CD31
VE-CADHERIN
CD31/VE-CAD/DAPI
H
NCRM4
I
J
K
HBMECs
bpECs
rBMECs
MW
25 kDa
20 kDa
50 kDa
37 kDa
22 kDa
p-CAV1
total CAV1
GAPDH
L
238kDa
171 kDa
41 kDa
31 kDa
200 kDa
ABCC4
M
P < 0.001
P = 0.030
tCAV1/GAPDH
N
P > 0.99
P = 0.0048
pCAV1/CAV1
O
P = 0.10
ABCC4/GAPDH
n.d.
P
endothelial cell monolayer
wound scratch
wound area
wound healing
Q
rBMECs untreated
rBMECs + angiogenic cocktail
Hours
0
6
12
18
24
30
36

**Figure S1. rBMECs derived from multiple hiPSC lines express BBB proteins related to tight junctions, transcellular transport and transporters. A)** Representative brightfield images show human induced pluripotent stem cells (hiPSC; IMR90 line) on select days (D1, D6 and D9) of the differentiation process, bpECs (post-sort A), and rBMECs (post-sort B). **B)** Dotted bar graph of the relative EpCAM protein surface expression in hiPSCs and rBMECs (IMR90). Mean ± SD, n = 4-6 from 2 independent differentiations, Welch's t-test. **C-D)** Dotted bar graphs of the percentage of $CD31^+$ and VE-$CADHERIN^+$ cells obtained from hiPSC lines (IMR90, FA0000010 and NCRM5) post-sort A (**C**; bpECs) and post-sort B (**D**; rBMECs). Each dot represents one independent differentiation. **E-G)** Representative immunofluorescence (IF) images of bpECs and rBMECs derived from the FA0000010 (FA10) hiPSC line for **(E)** CLAUDIN-5 (green) and ZO-1 (red), **(F)** OCCLUDIN (green), **(G)** CD31 (green) and VE-CADHERIN (red). DAPI (blue) labels nuclei in all merged images. Scale bar = 25 µm. **H-J)** Representative IF images of bpECs and rBMECs derived from the NCRM4 hiPSC line for **(H)** CLAUDIN-5 (green) and ZO-1 (red), **(I)** OCCLUDIN (green), **(J)** CD31 (green) and VE-CADHERIN (red). DAPI (blue) labels nuclei in all merged images. Scale bar = 25 µm. **K-L)** Representative western blots for phosphorylated CAVEOLIN-1 (pCAV1), total CAVEOLIN-1 (tCAV1), and ABCC4 proteins. GAPDH protein levels are used to normalize protein levels among different lanes. **M)** Quantification of the ratio of total-CAV1 protein over GAPDH protein levels. Mean ± SD, n = 8-9 from three independent differentiations, one-way ANOVA. **N)** Quantification for the ratio of phosphorylated CAV-1/CAV-1 protein levels. Mean ± SD, n = 8-9 from three independent differentiations, Welch's ANOVA. **O)** Quantification of the ratio of ABCC4 protein over GAPDH protein levels. Mean ± SD, n = 6 from two independent differentiations, unpaired t-test. **P)** Schematic of the wound scratch assay. **Q)** Representative images of the wound scratch assay in rBMECs over a 36-hour time period in the absence (left), or presence (right), of an angiogenic cocktail (see Methods for more details).

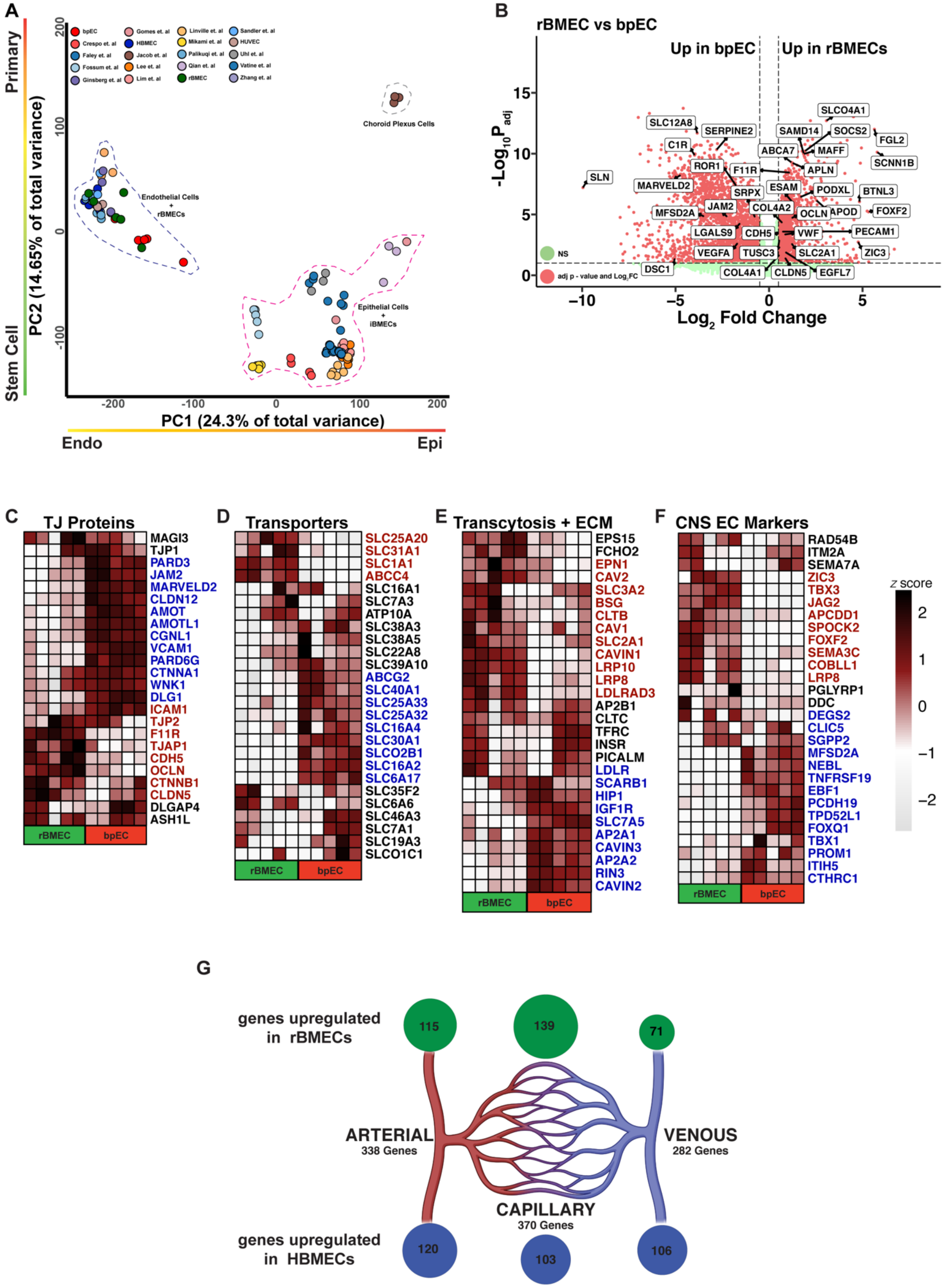
A
Primary
Stem Cell
PC2 (14.65% of total variance)
PC1 (24.3% of total variance)
Endo
Epi
bpEC
Crespo et. al
Faley et. al
Fossum et. al
Ginsberg et. al
Gomes et. al
HBMEC
Jacob et. al
Lee et. al
Lim et. al
Linville et. al
Mikami et. al
Palikuqi et. al
Qian et. al
rBMEC
Sandler et. al
HUVEC
Uhl et. al
Vatine et. al
Zhang et. al
Choroid Plexus Cells
Endothelial Cells + rBMECs
Epithelial Cells + iBMECs
B
rBMEC vs bpEC
Up in bpEC
Up in rBMECs
$-Log_{10} P_{adj}$
$Log_2$ Fold Change
NS
adj p - value and $Log_2 FC$
SLN
SLC12A8
SERPINE2
C1R
ROR1
F11R
MARVELD2
SRPX
MFSD2A
JAM2
LGALS9
VEGFA
DSC1
CDH5
TUSC3
COL4A1
COL4A2
ESAM
SLCO4A1
SAMD14
SOCS2
FGL2
ABCA7
MAFF
APLN
SCNN1B
PODXL
BTNL3
OCLN
APOD
FOXF2
VWF
PECAM1
SLC2A1
ZIC3
CLDN5
EGFL7
C
TJ Proteins
MAGI3
TJP1
PARD3
JAM2
MARVELD2
CLDN12
AMOT
AMOTL1
CGNL1
VCAM1
PARD6G
CTNNA1
WNK1
DLG1
ICAM1
TJP2
F11R
TJAP1
CDH5
OCLN
CTNNB1
CLDN5
DLGAP4
ASH1L
rBMEC
bpEC
D
Transporters
SLC25A20
SLC31A1
SLC1A1
ABCC4
SLC16A1
SLC7A3
ATP10A
SLC38A3
SLC38A5
SLC22A8
SLC39A10
ABCG2
SLC40A1
SLC25A33
SLC25A32
SLC16A4
SLC30A1
SLCO2B1
SLC16A2
SLC6A17
SLC35F2
SLC6A6
SLC46A3
SLC7A1
SLC19A3
SLCO1C1
rBMEC
bpEC
E
Transcytosis + ECM
EPS15
FCHO2
EPN1
CAV2
SLC3A2
BSG
CLTB
CAV1
SLC2A1
CAVIN1
LRP10
LRP8
LDLRAD3
AP2B1
CLTC
TFRC
INSR
PICALM
LDLR
SCARB1
HIP1
IGF1R
SLC7A5
AP2A1
CAVIN3
AP2A2
RIN3
CAVIN2
rBMEC
bpEC
F
CNS EC Markers
RAD54B
ITM2A
SEMA7A
ZIC3
TBX3
JAG2
APCDD1
SPOCK2
FOXF2
SEMA3C
COBLL1
LRP8
PGLYRP1
DDC
DEGS2
CLIC5
SGPP2
MFSD2A
NEBL
TNFRSF19
EBF1
PCDH19
TPD52L1
FOXQ1
TBX1
PROM1
ITIH5
CTHRC1
rBMEC
bpEC
z score
G
genes upregulated in rBMECs
115
139
71
ARTERIAL
338 Genes
VENOUS
282 Genes
CAPILLARY
370 Genes
genes upregulated in HBMECs
120
103
106

**Figure S2. The rBMEC transcriptome shows higher expression of some key BBB genes compared to the bpEC transcriptome. A)** PCA plot illustrating endothelial versus epithelial cell identity (PC1) and stem cell-derived versus primary cells (PC2) of 155 sequenced mRNA samples from several published studies and our study (see Results, Methods and References for more details about the published samples and the computational analysis performed for this PCA plot). **B)** Volcano plot showing the differentially expressed genes in rBMECs compared to bpECs. The genes listed on the right of the volcano plot are upregulated in rBMECs, and those on the left of the volcano plot are upregulated in bpECs. All listed BBB-specific genes are statistically significant ($p_{adj} < 0.05$, $|logFC| > 0.25$). **C-F)** Heatmaps of the relative expression of BBB-specific transcripts related to **(C)** tight junction genes, **(D)** transporter genes, **(E)** transcellular transport and ECM-related genes, and **(F)** CNS endothelial cell identity genes in bpECs and rBMECs. The scale is presented as log(z-score). The dark colors mean a high z-score and the light colors mean a low z-score. The upregulated genes in rBMECs are labelled in red text, and the upregulated genes in bpECs are labelled in blue text. The scale bar for all heatmaps is shown on the right. **G)** Schematic diagram of vascular zonation together with circles illustrating the number of upregulated genes in rBMECs (green) and HBMECs (blue) that are characteristic of a specific vascular zonation identity (arterial, capillary and venous). rBMECs cells do not have a specific zonation identity, as they express a similar number of arterial and capillary identity genes.

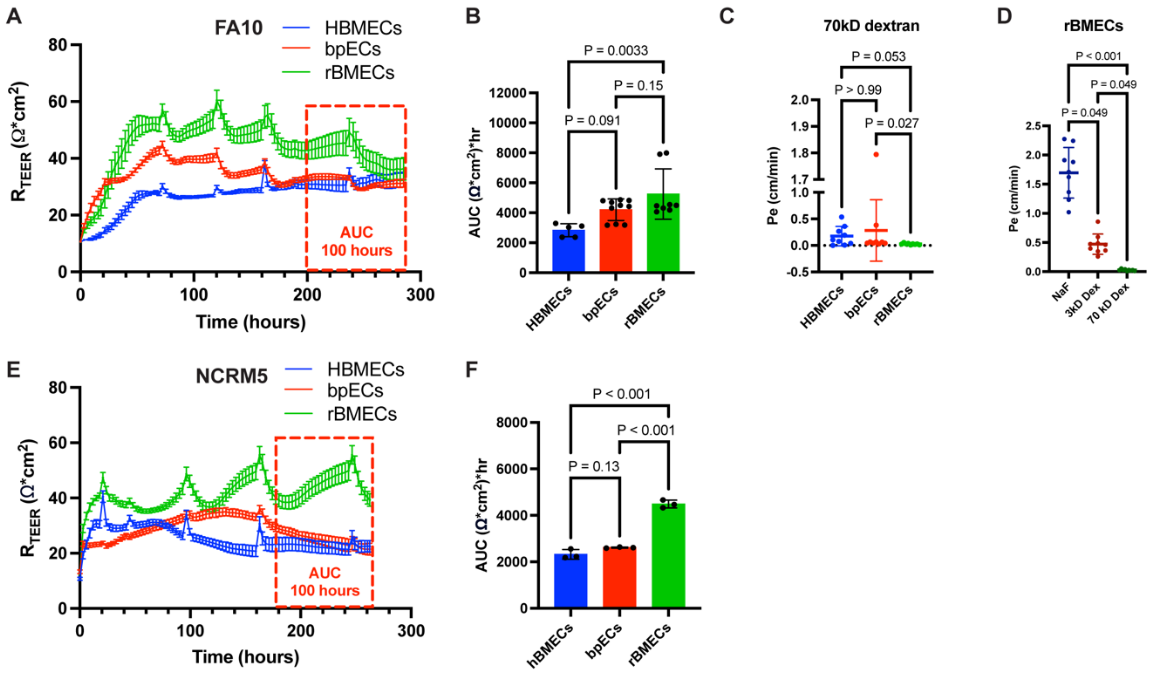


**Figure S3**. **rBMECs derived from multiple iPSC lines show robust functional barrier properties. A-B)** Representative TEER measurements obtained with an ECIS instrument over 280 hours and the dotted bar graph of the area under the curve (AUC) quantification (red box) for FA10 hiPSC-derived bpECs, rBMECs, and primary HBMECs. The red box indicates the period used for the AUC quantification. Mean ± SD, n = 5 - 10 from 3 independent differentiations, one-way ANOVA. **C)** Dotted bar graph of tracer permeability through an endothelial cell monolayer in the transwell assay using a 70 kDa dextran (Pe). Mean ± SD, n = 9 from 3 independent differentiations, Kruskal-Wallis test. **D)** Permeability (Pe) comparisons for sodium fluorescein (NaF; 332 Da), 3 and 70 kDa dextran tracers across rBMECs monolayers, Kruskal-Wallis test. Mean ± SD, n = 9 from 3 independent differentiations from IMR90 hiPSCs. **E-F)** Representative TEER measurements with the ECIS instruments and bar graph of the AUC quantification for NCRM-5 hiPSC-derived bpECs, rBMECs, and HBMECs. Mean ± SD, n = 3 from 1 independent differentiation, one-way ANOVA.

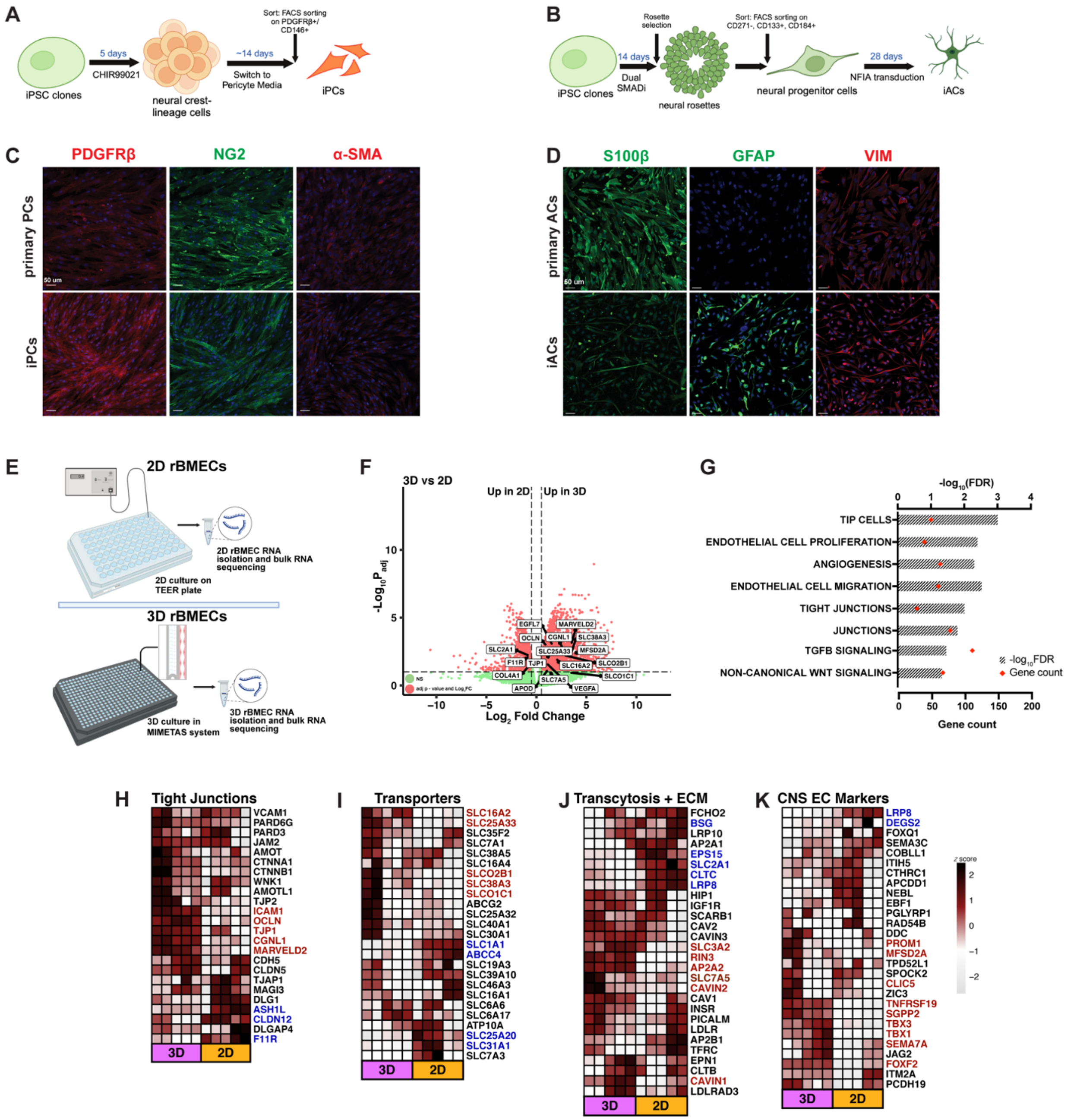


**Figure S4. Characterization of hiPSC-derived pericytes and astrocytes for the 3D NVU/BBB system, and upregulation of BBB relevant pathways in rBMECs after 3D microfluidic culture in the MIMETAS platform. A-B)** Schematic diagrams showing the strategy for generation of IMR90 hiPSC-derived pericytes and FA10 hiPSC-derived astrocytes. **C)** Representative immunofluorescence (IF) staining of primary and hiPSC-derived pericytes (iPCs) for PDGFRβ (red), NG2 (green), and α-SMA (green). **D)** Representative IF images of hiPSC-derived astrocytes (iACs) for S100β (green), GFAP (green) and VIMENTIN (red). **E)** Schematic diagram for the isolation, bulk mRNA sequencing and computational analysis of rBMEC samples from the 2D (monolayer) versus 3D (microfluidic tubules) MIMETAS system. **F)** Volcano plot highlighting significantly upregulated and downregulated BBB-specific genes in rBMECs cultured in the 3D system (right) compared to the 2D monolayer (left). **G)** Upregulated GSEA pathways in rBMECs cultured in the

3D system compared to the 2D monolayer. Red dots label gene counts, while striped bars indicate -log(FDR) values. **H-K)** Heatmap of BBB-specific genes (tight junction proteins, transporters, regulators of receptor-mediated transport, extracellular matrix, and CNS EC genes) upregulated and downregulated in rBMECs cultured in 3D versus 2D. The upregulated genes in 3D rBMECs are shown in red text and the upregulated genes in 2D rBMECs are shown in blue text. The scale is presented as log(Z score). The dark colors mean a high Z score and the light colors mean a low Z score.

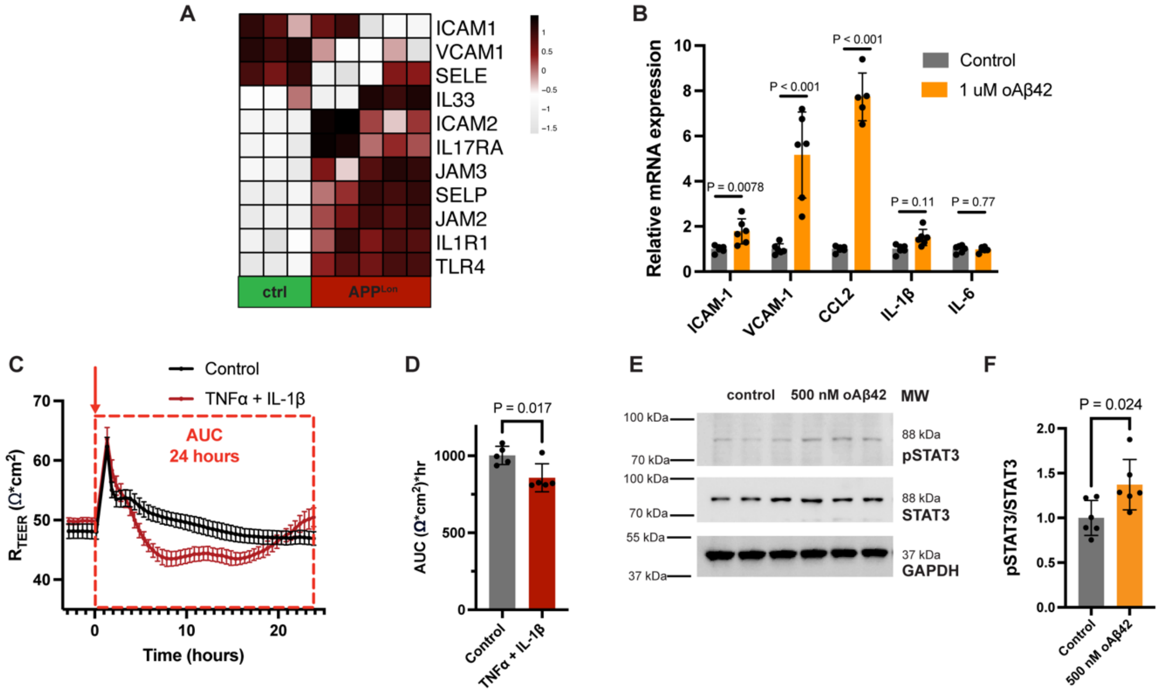


**Figure S5. Several neuroinflammatory markers are elevated in both *APP^Lon* rBMECs and oAβ42-treated control rBMECs. A)** Heatmap showing upregulated leukocyte adhesion molecules (LAMs) in *APP^Lon* rBMECs compared to control rBMECs. **B)** Bar graph of the relative expression of select inflammatory genes in IMR90-derived rBMECs by RT-qPCR after 72 hours of treatment with 1 uM oAβ42. Mean ± SD, n = 6 from 2 independent differentiations, unpaired t-test. **C-D)** Representative TEER measurements with the ECIS instrument and bar graph of the AUC quantification for IMR90-derived rBMECs treated with TNF-α (10 ng/mL) and IL-1β (10 ng/mL). Mean ± SD, n = 5 from 2 independent differentiations, unpaired t-test. **E-F)** Representative western blots and quantification of phosphorylated STAT3 and total STAT3 in IMR90-derived rBMECs after a 6-hour treatment with 500 nM oAβ42. Mean ± SD, n = 6 from 2 independent differentiations, unpaired t-test.

**Table S1. The list of primers used for the RT-PCR experiments described in Figures 5F and S5B.**

| Gene | Forward Primer | Reverse Primer |
|---|---|---|
| ICAM-1 | CAATGTGCTATTCAAACTGCCC | CAGCGTAGGGTAAGGTTCTTG |
| VCAM-1 | TCTACGCTGACAATGAATCCTG | AGGGCCACTCAAATGAATCTC |
| E-Selectin | AAGTTCGCCTGTCCTGAAG | CAGAAAGTCCAGCTACCAAGG |
| CCL2 | CCTCCAGCATGAAAGTCTCTG | TCTGCACTGAGATCTTCCTATTG |
| TNFα | ACTTTGGAGTGATCGGCC | GCTTGAGGGTTTGCTACAAC |
| IL-1β | ATGCACCTGTACGATCACTG | ACAAAGGACATGGAGAACACC |
| IL-6 | CAACCTGAACCTTCCAAAGAT | ACCTCAAACTCCAAAAGACCAG |
| GAPDH | TGAAGGTCGGAGTCAACGGATTTGG | CATGTAGGCCATGAGGTCCACCAC |

**Video S1 (separate file).** 3D projection of rBMECs in the MIMETAS platform after IF staining and imaging with a Zeiss LSM900 confocal microscope. The rBMECs are stained with antibodies for VE-CADHERIN (green) and CD31 (red), associated with **Figure 4C**.

**Video S2 (separate file).** 3D projection of rBMECs in the MIMETAS platform after IF staining and imaging with a Zeiss LSM900 confocal microscope. The rBMECs are stained with antibodies for ZO-1 (red) and CLAUDIN-5 (green), associated with **Figure 4C**.

**Video S3 (separate file).** 3D NVU showing iPCs (PDGFRβ$^+$ cells) and iACs (S100β$^+$ cells) migrating from the brain channel towards the blood channel through the ECM "middle" channel associated with **Figure 4D**.

**Video S4 (separate file).** 3D projection of rBMECs, iPCs, and iACs in the MIMETAS platform after IF staining and imaging with a Zeiss LSM900 confocal microscope. The cells are stained with antibodies for ZO-1 (red, rBMECs), S100β (green, iACs), and PDGFRβ (blue, iPCs), associated with **Figure 4E**.

**Dataset S1 (separate file). Bulk RNA sequencing analysis of rBMECs versus bpECs and HBMECs. A)** PCA components for comparison of endothelial cells from multiple organs. The first fifty PCs are shown. **B)** PCA components for brain endothelial cells only. **C)** PCA components for iPSC-derived brain endothelial cells. **D)** Differentially expressed genes between rBMECs and HBMECs. **E)** Differentially expressed GO terms between rBMECs and HBMECs. **F)** Differentially expressed genes between rBMECs and bpECs. **G)** Differentially expressed genes between rBMECs cultured in the 3D system versus the 2D monolayer. **H)** Gene list for GSEA analysis. **I)** Differentially expressed zonation genes in rBMECs and HBMECs. **J)** Differentially expressed genes between *APP*$^{Lon}$ rBMECs and *APP*$^{Ctrl}$ rBMECs**. K)** Differentially expressed GO pathways between *APP*$^{Lon}$ rBMECs and *APP*$^{Ctrl}$ rBMECs**.**

# Major Resources Table

## Antibodies

| Target antigen | Vendor or Source | Catalog # | Working concentration | Persistent ID / URL |
|---|---|---|---|---|
| Mouse monoclonal Anti-Human CD31 antibody (Clone JC70A) | Agilent | Cat#M082301-2 | 2 ug/mL | RRID: AB_2114471 |
| Rabbit polyclonal VE-Cadherin antibody | Abcam | Cat#ab33168 | 5 ug/mL | RRID: AB_870662 |
| Rabbit polyclonal anti-GAPDH antibody | Sigma | Cat#G9545 | 0.2 ug/mL | RRID: AB_796208 |
| Mouse monoclonal anti-S100β | Sigma-Aldrich | Cat#S2532 | 50 ug/mL | RRID: AB_477499 |
| Rabbit monoclonal anti-VCAM-1 | Abcam | Cat#ab134047 | 0.4 ug/mL | RRID: AB_2721053 |
| Rabbit monoclonal anti-ICAM-1 | Cell Signaling | Cat#67836T | 0.1 ug/mL | RRID: AB_2799738 |
| Mouse monoclonal anti-GFAP | EMD Millipore | Cat#MAB360 | 20 ug/mL | RRID: AB_11212597 |
| Rat Monoclonal anti-Ki-67 Antibody (SolA15) | Thermo Fisher | Cat#14-5698-82 | 1 ug/mL | RRID: AB_10854564 |
| Mouse monoclonal anti-Claudin-5 | Thermo Fisher | Cat#35-250-0 | IF: 2.5 ug/mL; WB: 0.5 ug/mL | RRID: AB_2533200 |
| Rabbit polyclonal anti-ZO-1 | Thermo Fisher | Cat#61-7300 | 1.25 ug/mL | RRID: AB_2533938 |
| Mouse monoclonal anti-Occludin | Thermo Fisher | Cat#33-1500 | IF: 2.5 ug/mL; WB: 0.5 ug/mL | RRID: AB_2533101 |
| Rabbit monoclonal anti-total Caveolin-1 | Sigma Aldrich | Cat#C4490 | 1.6 ug/mL | RRID: AB_262110 |
| Rabbit polyclonal anti-Phospho-Caveolin-1 | Cell Signaling | Cat#3251 | 0.04 ug/mL | RRID:AB_2244199 |
| Rabbit monoclonal anti-ABCC4/MRP4 | Cell Signaling | Cat#12857 | 0.01 ug/mL | RRID: AB_2798046 |
| Mouse PhosphoPair STAT3 (Tyr705) Antibody Set | Biolegend | Cat#699952 | 0.5 μg/mL | RRIDs: AB_2861053, AB_10897947 |
| Goat polyclonal PDGFRβ Antibody | R&D | Cat#AF385 | 2 ug/mL | RRID: AB_355339 |

| | | | | |
|---|---|---|---|---|
| Mouse monoclonal α-Smooth Muscle antibody | Sigma Aldrich | Cat#A5228 | 2.8 ug/mL | RRID: AB_262054 |
| Rabbit monoclonal anti-Vimentin antibody | Cell Signaling Technology | Cat#5741 | 0.2 ug/mL | RRID: AB_10695459 |
| Mouse monoclonal Neural/Glial Antigen 2 (NG2) Antibody (9.2.27), eBioscience™ | Thermo Fisher | Cat#14-6504-82 | 5 ug/mL | RRID: AB_10870987 |
| Mouse monoclonal anti-EpCAM Direct Conjugated Antibody, Alexa Fluor 488 | BioLegend | Cat#324210 | 1 ug/mL | RRID: AB_756084 |
| Mouse monoclonal anti-PECAM-1 Direct Conjugated Antibody, FITC | BioLegend | Cat#303104 | 2 ug/mL | RRID: AB_314330 |
| Recombinant human monoclonal anti-VE-Cadherin Direct Conjugated antibody, APC | Miltenyi Biotech | Cat#130-125-985 | 0.5 ug/mL | RRID: AB_2655162 |
| Mouse monoclonal anti-ICAM-1 Direct Conjugated Antibody, FITC | BioLegend | Cat#353107 | 4 ug/mL | RRID: AB_10898317 |
| Mouse monoclonal anti-VCAM-1 Direct Conjugated Antibody, APC | Biolegend | Cat#305809 | 1 ug/mL | RRID: AB_2304307 |
| Mouse monoclonal anti-CD184 Direct Conjugated Antibody, APC | BioLegend | Cat#306509 | 2 ug/mL | RRID: AB_314615 |
| Mouse monoclonal anti-CD133 Direct Conjugated Antibody, PE | BioLegend | Cat#372803 | 2 ug/ml | RRID: AB_2632879 |
| Mouse monoclonal anti-CD271 Direct Conjugated Antibody, FITC | BioLegend | Cat#345103 | 3 ug/mL | RRID: AB_1937226 |
| PE anti-human CD140b (PDGFRβ) Antibody | BioLegend | Cat#323605 | 1 ug/mL | RRID: AB_2299493 |
| PE/Cyanine7 anti-human CD146 Antibody | BioLegend | Cat#361008 | 0.5 ug/mL | RRID: AB_2562983 |

## DNA/cDNA Clones

| Clone Name | Sequence | Source / Repository | Persistent ID / URL |
|---|---|---|---|
| FOXF2 lentiviral construct | NM_001452.2 | This manuscript | Backbone: Addgene_41392 |
| ZIC3 lentiviral construct | NM_003413.4 | This manuscript | Backbone: Addgene_41392 |

## Cultured Cells

| Name | Vendor or Source | Sex (F, M, or unknown) | Persistent ID / URL |
|---|---|---|---|
| HEK293T | ATCC | unknown | Cat#CRL-1573 |
| Irradiated CF1 mouse embryonic fibroblasts | Thermo Fisher | unknown | Cat#A34181 |
| Primary Human Umbilical Vein Endothelial Cells | ATCC | unknown | Cat#PCS-100-010 |
| Primary Human Brain Microvascular Cells | Cell System | unknown | Cat#ACBRI376 |
| Human Brain Vascular Pericytes | ScienCell | unknown | Cat#1200 |
| Human Astrocytes | ScienCell | unknown | Cat#1800 |
| iPS(IMR90)-4 | WiCell | female | RUID# WISCi004-B |
| FA0000010 (FA10) | RUCDR | male | RUID# CUIMCi001-A |
| ND50025 (NCRM-4) | RUCDR/NINDS Collection | female | RUID#CR0000004, Clone ID: R149482761 |
| **ND50031 (NCRM-5)** | RUCDR/NINDS Collection | male | RUID#CR0000005, Clone ID: R149506778 |

## Data & Code Availability

| Description | Source / Repository | Persistent ID / URL |
|---|---|---|
| Bulk RNA sequencing data | this manuscript / NIH GEO | GSE267498 |
| | | |
| | | |

## Other

| Description | Source / Repository | Persistent ID / URL |
|---|---|---|
| **Chemicals, Peptides, and Recombinant Proteins** | | |
| Recombinant human FGF-2 | R&D Systems | Cat#3718-FB |

| | | |
|---|---|---|
| Recombinant human VEGF-165 | R&D Systems | Cat#293-VE-050/CF |
| Recombinant human VEGF-165 | Stem Cells Tech. | Cat#78073.1 |
| Recombinant human bFGF | Peprotech | Cat#100-18C-100ug |
| Recombinant human HGF | Peprotech | Cat#100-39H |
| Recombinant human Thymosinβ4 | R&D Systems | Cat#3390/100U |
| Recombinant human BMP-4 | R&D Systems | Cat#314-BP-500/CF |
| Recombinant β-amyloid (1-42) | rPeptide | Cat#A-1163-2 |
| Recombinant human TNFα | R&D Systems | 210-TA-020/CF |
| Recombinant human IL-1β | R&D Systems | 201-LB-005/CF |
| Human transferrin | Sigma-Aldrich | Cat#T3309 |
| Sodium fluorescein | Sigma-Aldrich | Cat#46960-25G-F |
| Dextran, 3 kDa, Alexa Fluor 680 | Thermo Fisher | Cat#D34681 |
| Dextran, 70 kDa, CF770 | Biotium | Cat#80123 |
| Collagen from human placenta, type IV | Sigma-Aldrich | Cat#C5533 |
| Fibronectin from human plasma | Sigma-Aldrich | Cat#F2006 |
| Cultrex Stem Cell Qualified RGF Basement Membrane | R&D | Cat#3434-010-02 |
| Insulin solution | Sigma-Aldrich | Cat#19278 |
| Puromycin | Sigma-Aldrich | Cat#P8833 |
| Poly-D-Lysine | MP Biomedicals | Cat#0215017510 |
| SB 431542 | Selleck Chemicals | Cat#S1067 |
| CHIR 99021 | Tocris | Cat #4423 |
| Collagenase Type IV | StemCell Technologies | Cat#07909 |
| Plasma derived-platelet poor serum | Sigma-Aldrich | Cat#P2918 |
| Knockout serum replacement | Thermo Fisher | Cat#10828028 |
| Fetal Bovine Serum, heat-inactivated | Sigma Aldrich | Cat#12306C |
| Bovine serum albumin | Sigma-Aldrich | Cat#A9576 |

| | | |
|---|---|---|
| Bovine serum albumin | Sigma-Aldrich | Cat#A9418 |
| Accutase | Thermo Fisher | Cat#A11105-01 |
| ReleSR | StemCell Technologies | Cat#100-0483 |
| Trypsin-EDTA (0.05%) | Thermo Fisher | Cat#25300-054 |
| β-mercaptoethanol | Thermo Fisher | Cat#21985-023 |
| GlutaMAX | Thermo Fisher | Cat#35050-061 |
| MEM non-essential amino acids | Thermo Fisher | Cat#11140-050 |
| Penicillin-streptomycin (10,000 U/mL) | Thermo Fisher | Cat#15140-122 |
| B-27 Supplement, minus vitamin A | Gibco | Cat#12-587-010 |
| N-2 Supplement | Gibco | Cat#17502048 |
| Rhodamine 123, FluoroPure™ grade | Invitrogen | Cat#R22420 |
| Bovine Serum Albumin (BSA), Alexa Fluor™ 594 conjugate | Invitrogen | Cat#A13101 |
| PSC-833 | Tocris | Cat#4042 |
| SuperBlock Blocking Buffer in PBS | Thermo Fisher | Cat#37515 |
| Triton-X | Thermo Fisher | Cat#A16046 |
| Tween 20 | Thermo Fisher | Cat#BP337 |
| SuperBlock T20 Blocking Buffer | Thermo Fisher | Cat#37536 |
| CF® Dye Biocytin CF640 | Biotium | Cat#92007 |
| CellTag 700 Stain | LI-COR | Cat#926-41090 |
| eBioscience Fixable Viability Dye eFluor 780 | Thermo Fisher | Cat#65-0865-14 |
| **Critical commercial assays** | | |
| BCA Protein Assay Kit | Millipore Sigma | Cat#71285-3 |
| SuperSignal West Pico PLUS Chemiluminescent Substrate | Thermo Fisher | Cat#34577 |
| RNeasy Mini Kit | Qiagen | Cat#74104 |

| | | |
|---|---|---|
| RNeasy Micro Kit | Qiagen | Cat#74004 |
| Maxima First Strand cDNA Synthesis Kit for RT-qPCR | Thermo Fisher | Cat#K1642 |
| Lipofectamine 3000 Transfection Kit | Thermo Fisher | Cat#L3000001 |
| 12mm diameter, 12μm pore transwell insert | Corning | Cat#3401 |
| Human endothelial serum-free media | Gibco | Cat#11111044 |
| Endothelial Cell Growth Medium MV2 | PromoCell | Cat#C-22121 |
| Astrocyte Medium Kit | ScienCell | Cat#1801 |
| Pericyte Medium Kit | ScienCell | Cat#1201 |
| F-12, phenol red free | R&D | Cat#M25350 |
| DMEM / F12 (1:1) | Gibco | Cat#11320-033 |
| F12 | Gibco | Cat#11765-054 |
| IMDM | Gibco | Cat#12440-053 |
| DMEM | Gibco | Cat#11965-092 |
| Knockout DMEM | Gibco | Cat#10829-018 |
