# Supplementary material for: Generation of hiPSC-Derived Brain Microvascular Endothelial Cells Using Directed Differentiation and Transcriptional Reprogramming
Source: Arterioscler Thromb Vasc Biol. 2025 Nov 25;46(1):210–31. doi: 10.1161/ATVBAHA.125.323397 (PMC12721698; doi:10.1161/ATVBAHA.125.323397)
Supplement: Supplementary file 2 [file atv-46-210-s002.pdf]

CLAUDIN-5 (Figure 1F/G)

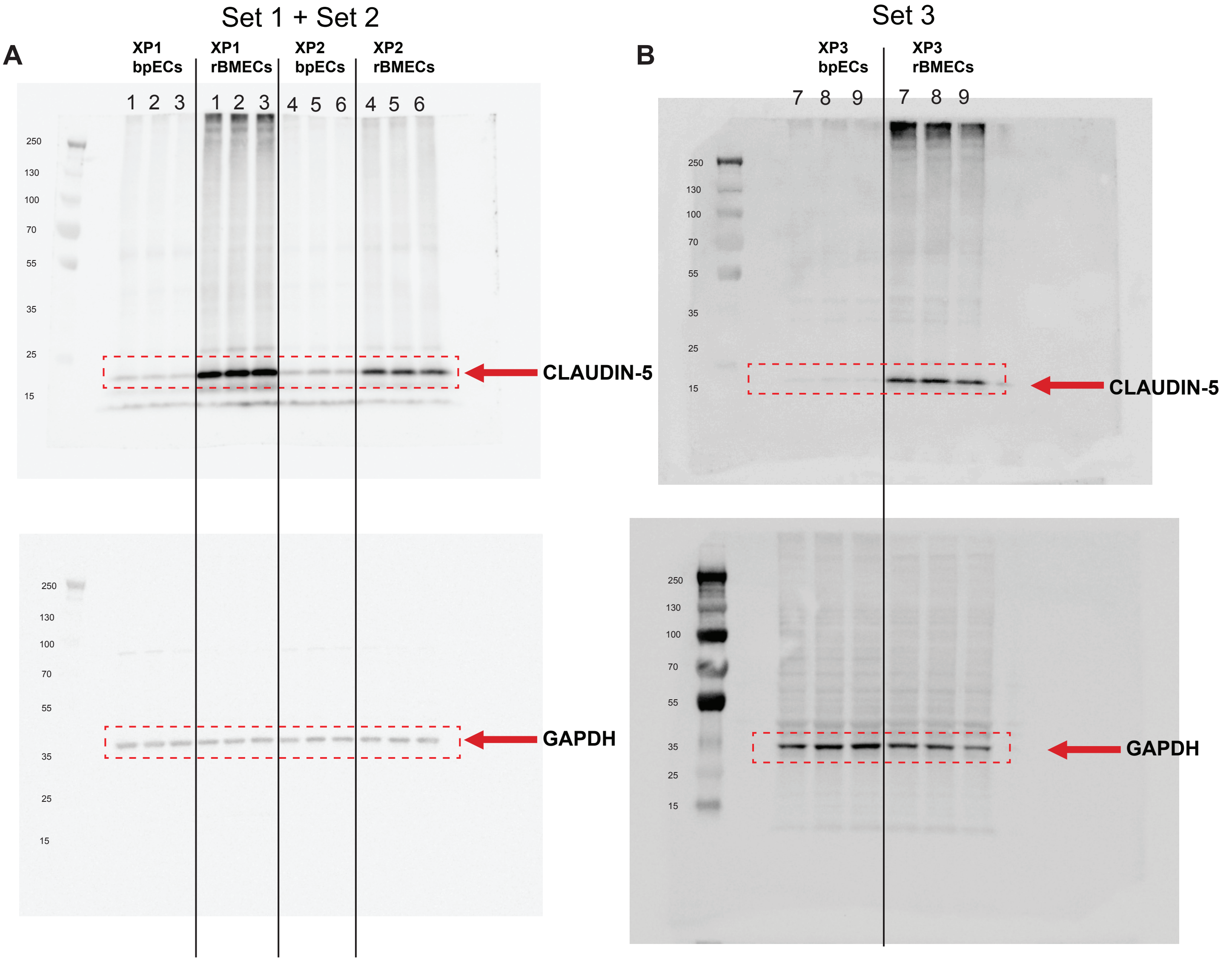

Representative gel used in Figure 1F (6 lanes on left)

OCCLUDIN (Figure 1F/G)

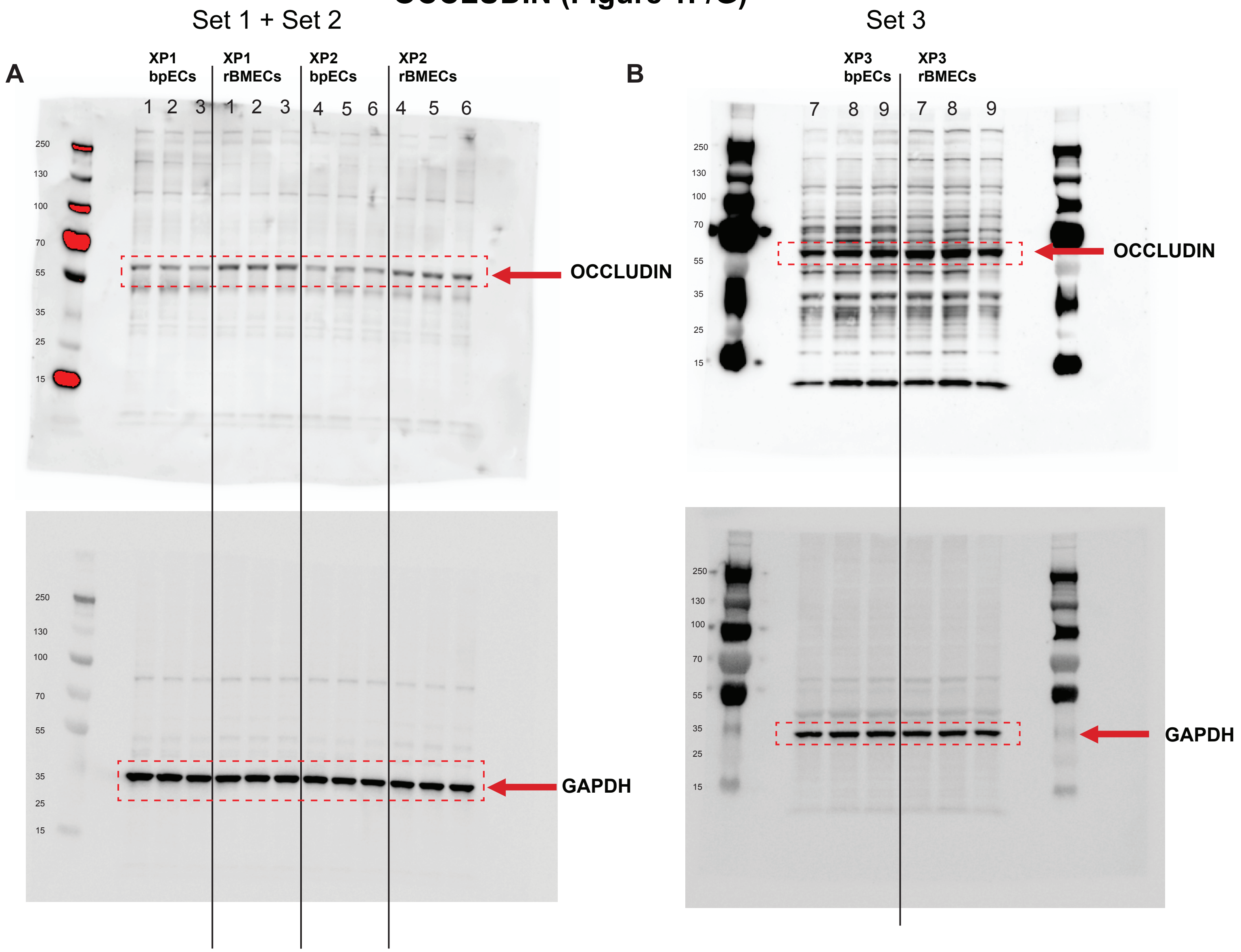

Representative gel
used in Figure 1F
(6 lanes on left)

VCAM-1 (Figure 5H/I)

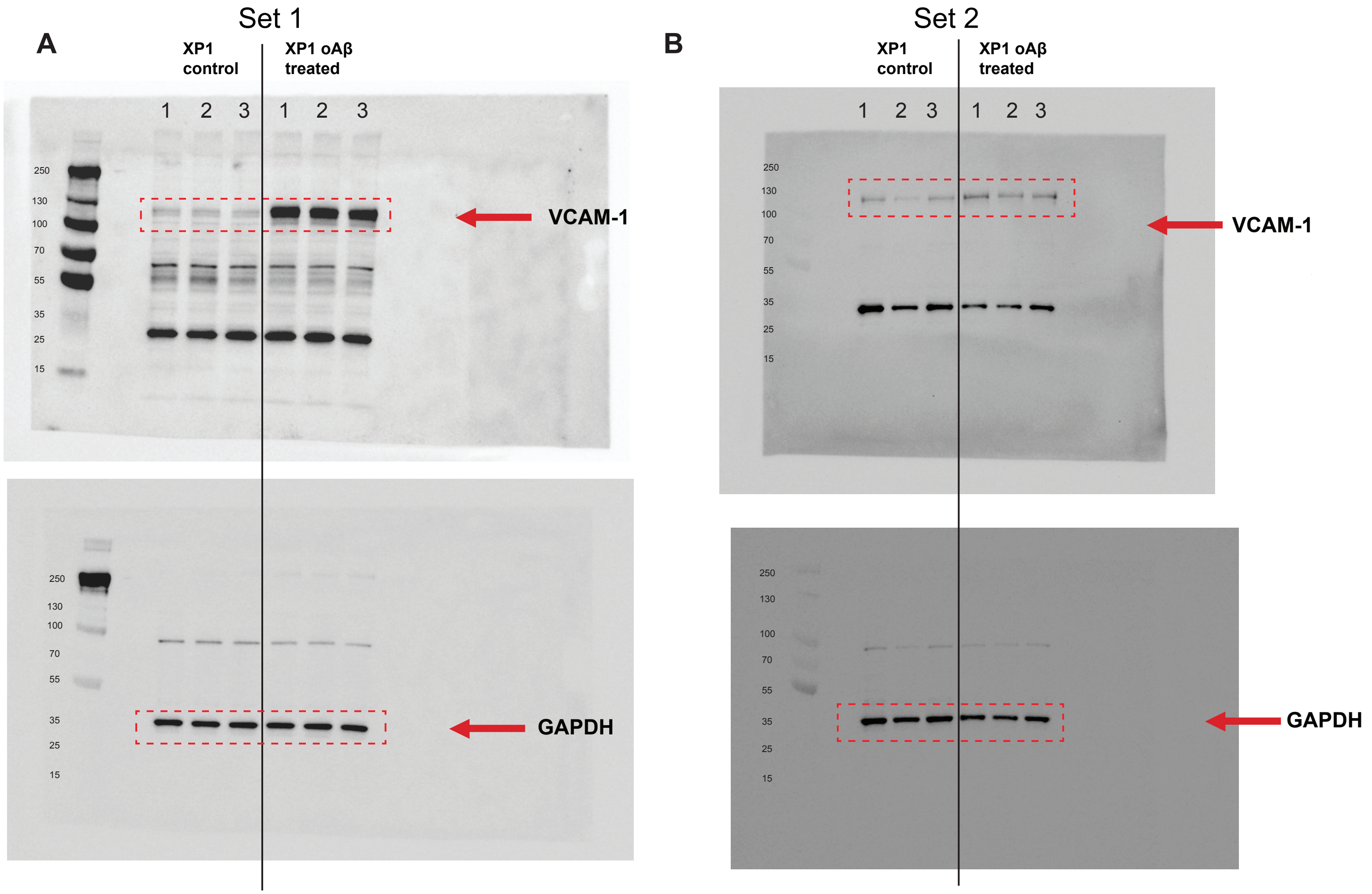

Representative gel used in Figure 5H

A

ICAM-1 (Figure 5H/I)

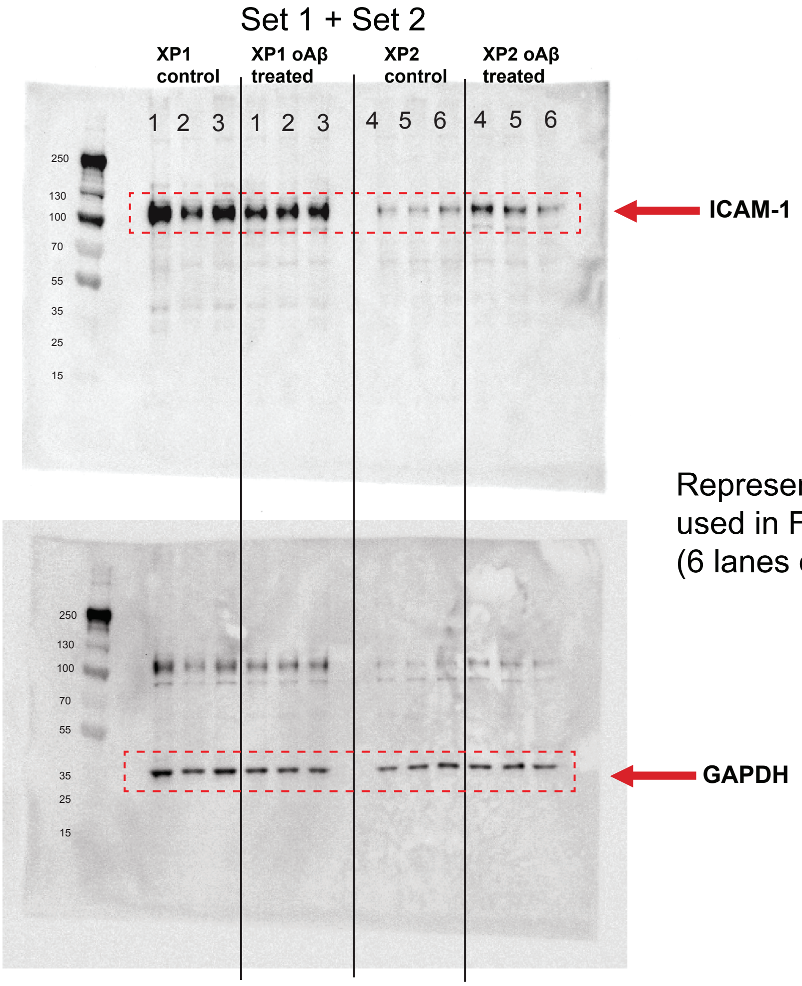

Representative gel used in Figure 5H (6 lanes on right)

total CAV1/pCAV1 (Figure S1K, M-N)

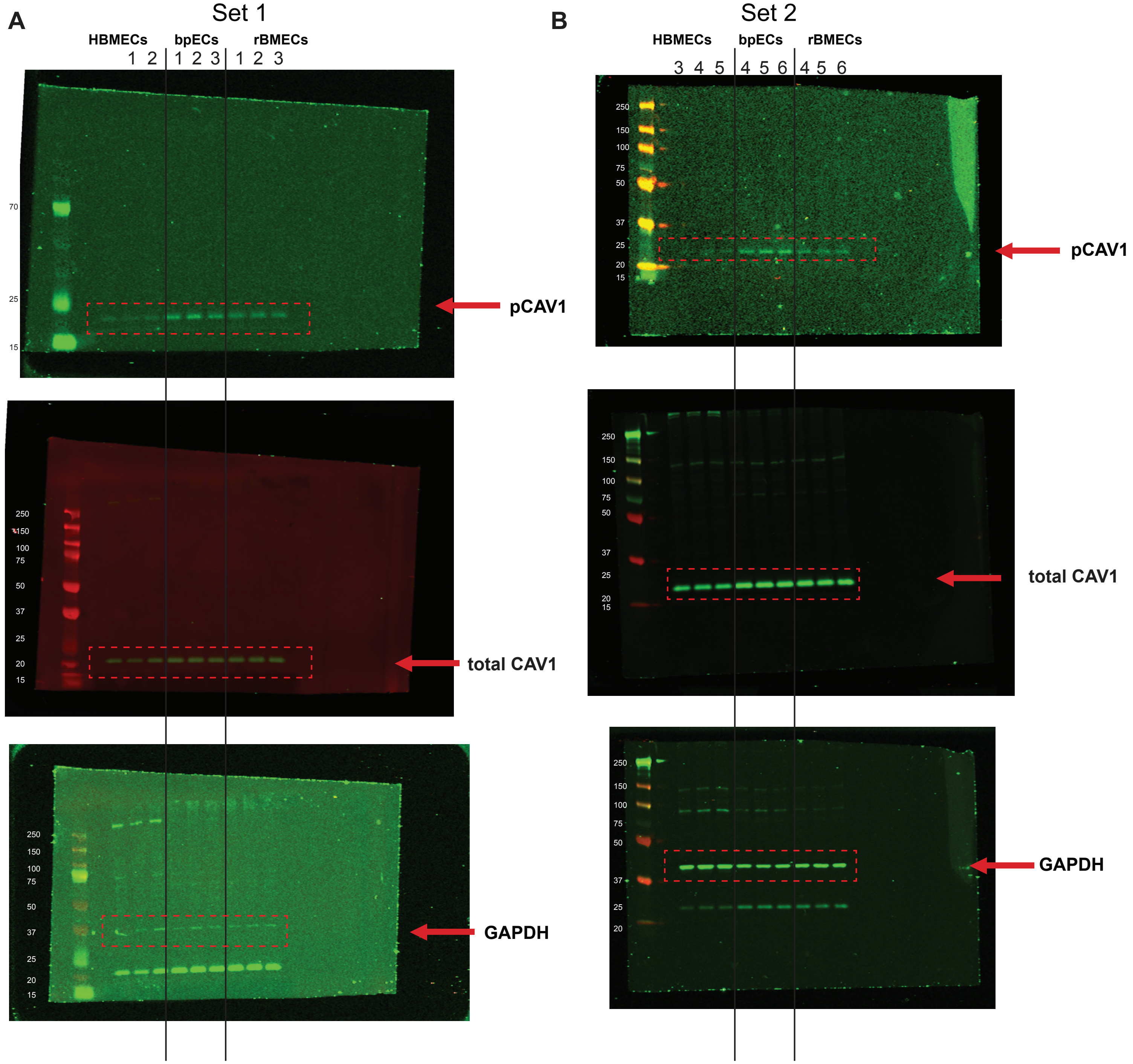

Note: The left-most lane for HBMECs was not quantified due to band issue.

Representative gel used in Figure S1K

total CAV1/pCAV1 (Figure S1K, M-N)

C

Set 3

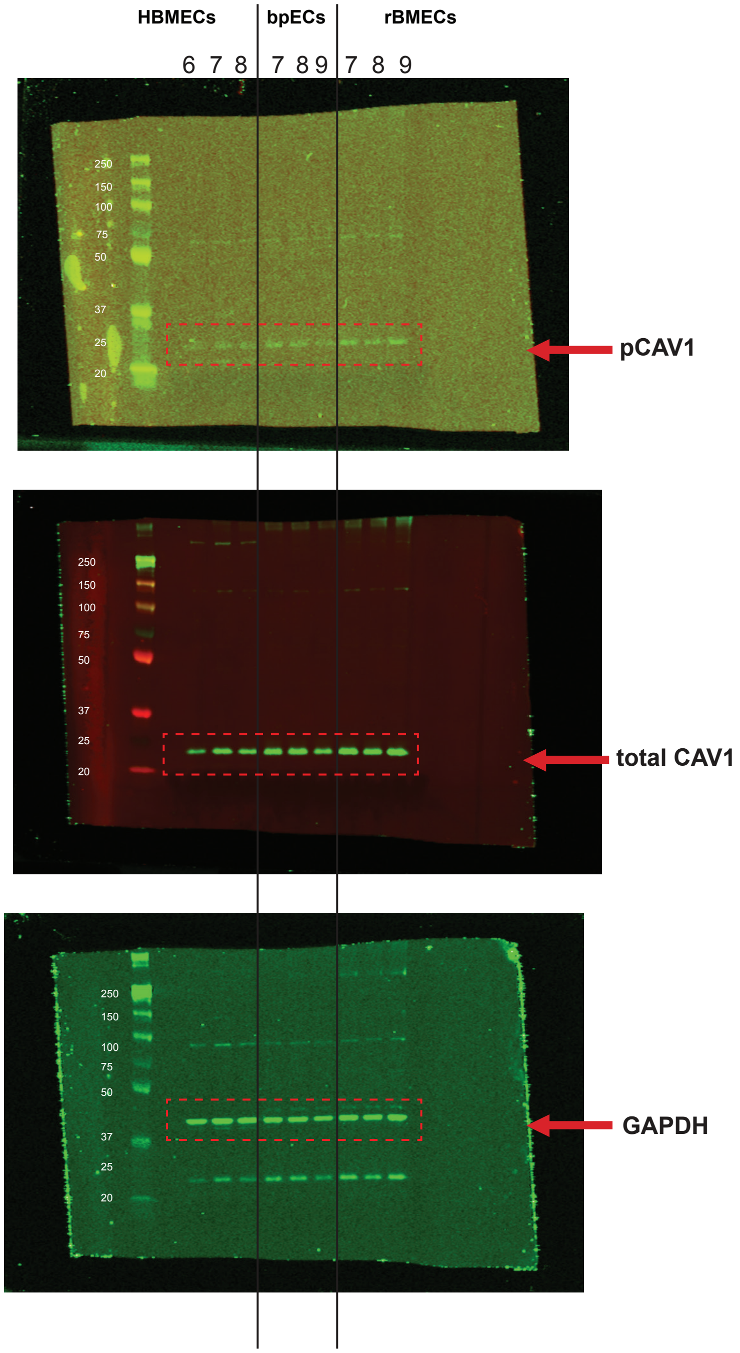

ABCC4 (Figure S1L/O)

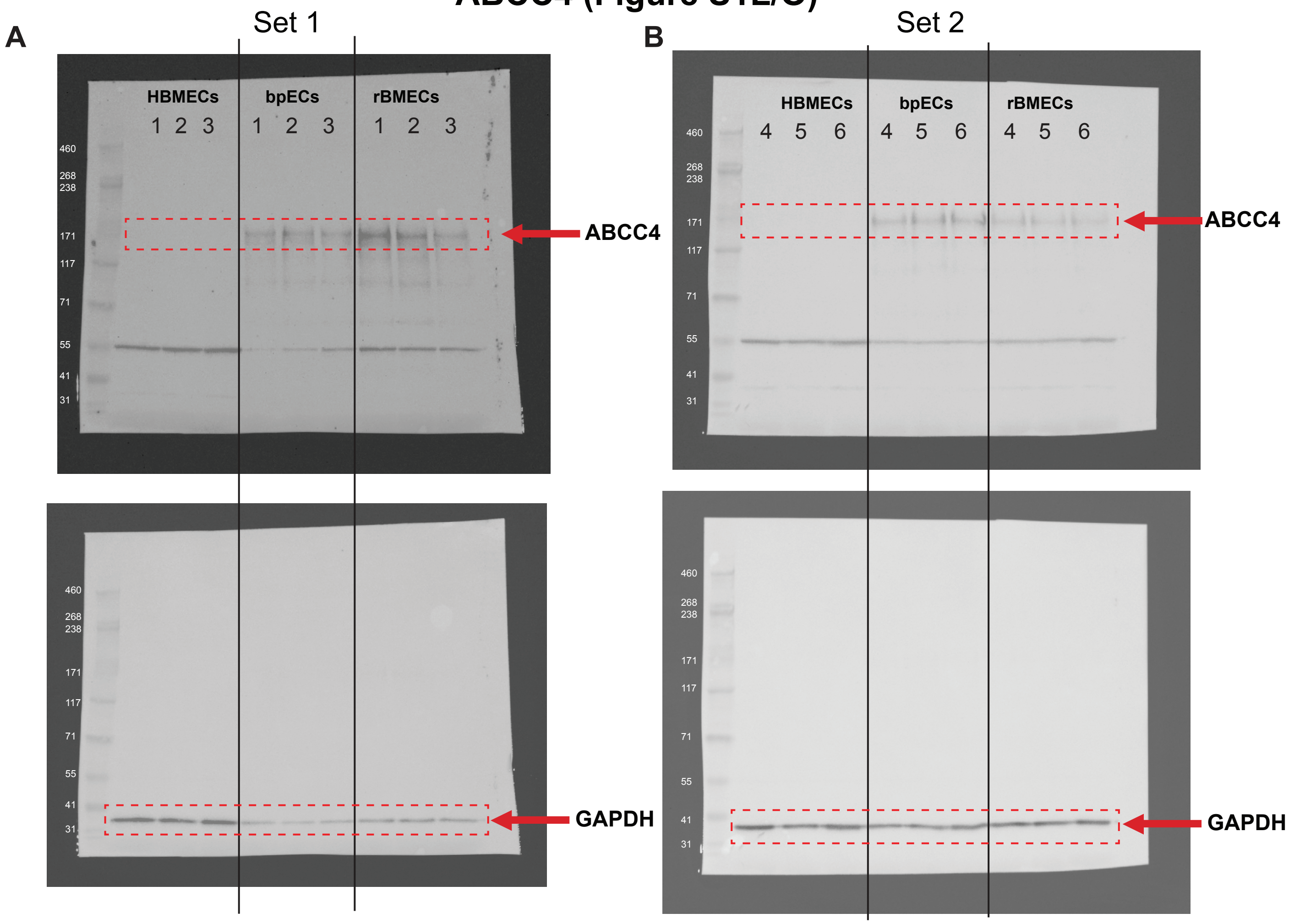

Representative gel used in Figure S1L

STAT3/pSTAT3 (Figure S5E-F)

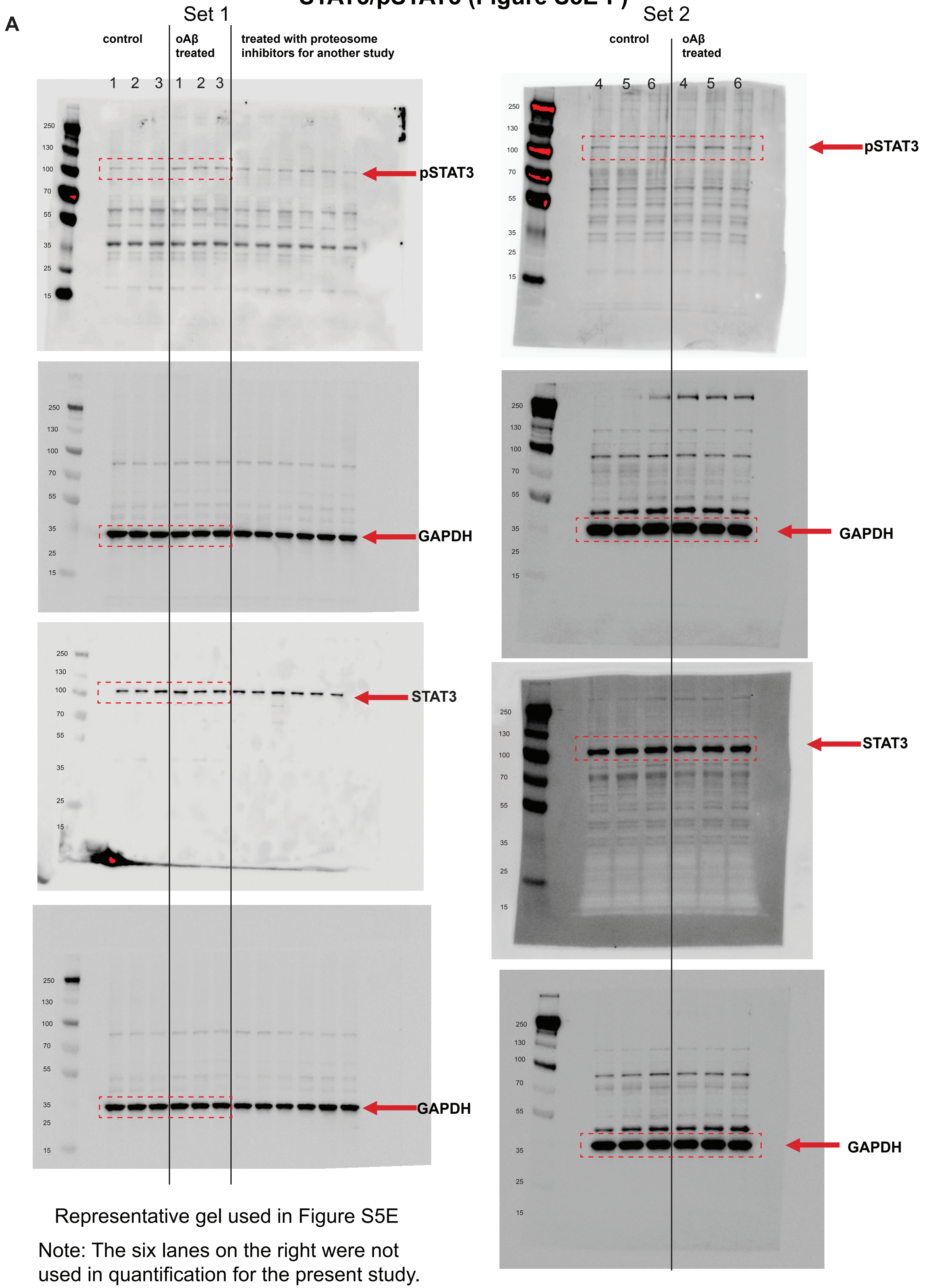

Representative gel used in Figure S5E

Note: The six lanes on the right were not used in quantification for the present study.
